# More bones of *Leptoptilos robustus* from Flores reveal new insights into giant marabou stork paleobiology and biogeography

**DOI:** 10.1098/rsos.220435

**Published:** 2022-07-13

**Authors:** Hanneke J. M. Meijer, Thomas Sutikna, E. Wahyu Saptomo, Matthew W. Tocheri

**Affiliations:** ^1^ Department of Natural History, University Museum, University of Bergen, 5007 Bergen, Norway; ^2^ Human Origins Program, National Museum of Natural History, Smithsonian Institution, Washington DC 20013, USA; ^3^ Naturalis Biodiversity Center, Leiden, The Netherlands; ^4^ Australian Research Council Centre of Excellence for Australian Biodiversity and Heritage, University of Wollongong, Wollongong, New South Wales 2522, Australia; ^5^ Centre for Archaeological Science, School of Earth and Environmental Sciences, University of Wollongong, Wollongong, New South Wales 2522, Australia; ^6^ Pusat Penelitian Arkeologi Nasional, Jakarta 12510, Indonesia; ^7^ Department of Anthropology, Lakehead University, Thunder Bay, Ontario P7B 5E1, Canada

**Keywords:** giant stork, Flores, Liang Bua, Late Pleistocene

## Abstract

Liang Bua (Flores, Indonesia) has yielded remains of a faunal community that included small-bodied and small-brained hominins, dwarf proboscideans, Komodo dragons, vultures and giant marabou storks (*Leptoptilos robustus*). Previous research suggested that *L. robustus* evolved from a smaller *Leptoptilos* *dubius*-like Middle Pleistocene ancestor and may have been flightless. However, analyses of this species' considerably expanded hypodigm (*n* = 43, MNI = 5), which includes 21 newly discovered bones described here for the first time, reveals that the wing bones of *L. robustus* were well-developed and this species was almost certainly capable of active flight. Moreover, *L. robustus* bones are broadly similar to *Leptoptilos falconeri* remains from sites in Africa and Eurasia, and its overall size range is comparable to fossils attributed to *L. falconeri* and similar specimens, as well as those of *Leptoptilos lüi* (China) and *Leptoptilos titan* (Java). This suggests that a Pleistocene dispersal of *L. falconeri* into Island Southeast Asia may have given rise to populations of giant marabou storks in this region. As *L. robustus* and *L. titan* are the most recent known representatives of these once plentiful giant marabou storks, Island Southeast Asia likely acted as a refugium for the last surviving members of this lineage.

## 1. Introduction

Extinct giant marabou stork species were broadly distributed across continental Africa and Eurasia during the Plio-Pleistocene [1–3]. Of these, *Leptoptilos falconeri* is the best known with fossils of this species recovered at sites in Africa (Pliocene-Early Pleistocene) and Eurasia (Pliocene) [1]. Relatively younger sites in northeastern China and Java preserve evidence of *Leptoptilos lüi* (approx. 260 000 years ago at Jinniushan) [2] and *Leptoptilos titan* (Late Pleistocene at Watualang) [4], respectively. In some instances, these giant carnivorous birds have been found in association with proboscideans, vultures, and even hominins, suggesting a possible symbiotic relationship existed among these taxa [2,3]. The emergence and expansion of grasslands in East Africa during the Late Pliocene likely facilitated the dispersal of large mammalian species from Africa into Asia (and vice versa), and subsequently during the Pleistocene from mainland Asia into Southeast Asia [5–7]. As opportunistic scavengers, giant marabou storks and vultures almost certainly would have also dispersed along with their primary sources of food (i.e. large mammal carcasses).

**Figure 1.**
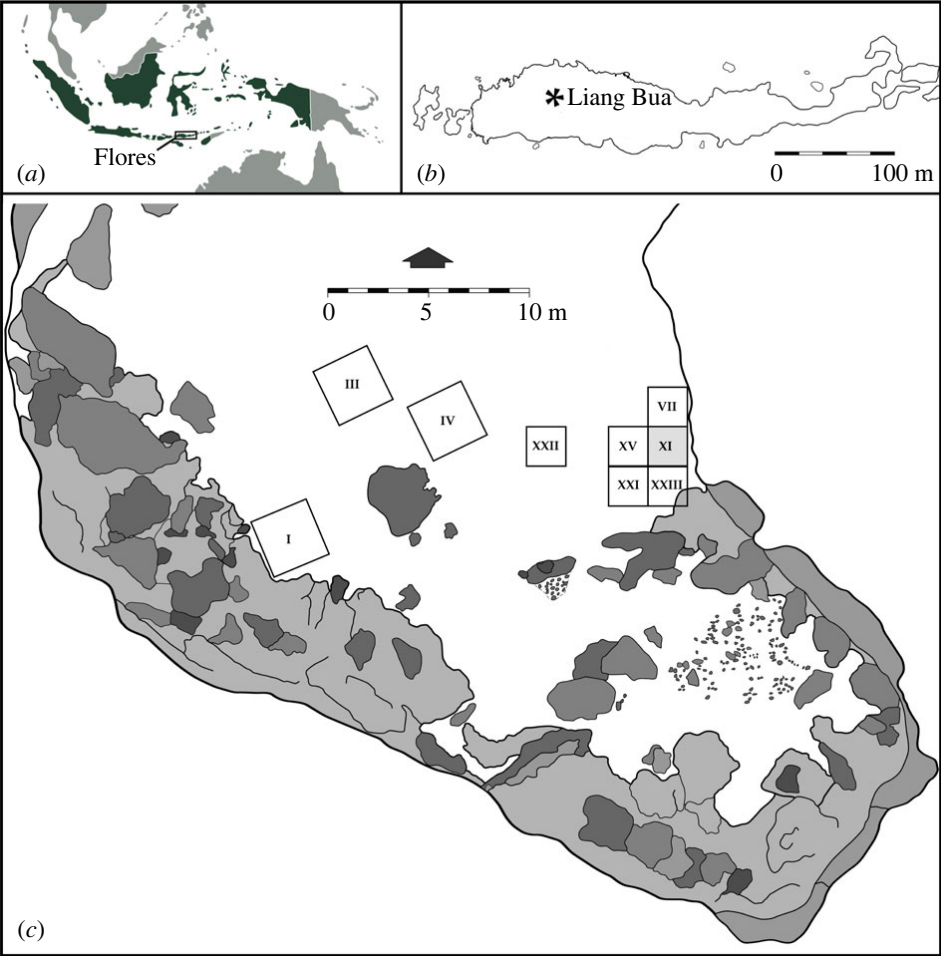
Site location: (*a*), location of Flores in Indonesia; (*b*), location of Liang Bua on Flores; and (*c*), plan of Liang Bua showing the excavated squares that have yielded the skeletal elements of *L*. *robustus* that are the focus of the present study. All previously published descriptions of *L*. *robustus* derive solely from material recovered from Sector XI (shown in grey).

Adding to this interesting story is yet another extinct giant marabou stork species, *Leptoptilos robustus*, discovered on the Indonesian island of Flores, an oceanic island that is part of Wallacea and has never been connected to either the Asian or Australian continental land masses [8]. Found in Late Pleistocene sediments (approx. 100–60 ka) of the limestone cave Liang Bua (figure 1), *L. robustus* is associated with a limited number of species with body masses greater than approximately 3 kg, including a dwarf proboscidean (*Stegodon florensis insularis*), large varanid (*Varanus komodoensis*) and vulture (*Trigonoceps* sp.), as well as a small-bodied and small-brained hominin (*Homo floresiensis*) [8–19].

First described by Meijer and Awe Due [8] based on four bones—a left ulna (distal portion), left carpometacarpus (proximal portion), left tibiotarsus (distal portion) and relatively complete left femur—*L. robustus* was estimated to have weighed approximately 16 kg, larger than any extant *Leptoptilos* species. It was interpreted to have evolved from a smaller *L*. *dubius*-like Middle Pleistocene ancestor and the relatively thick cortical bone wall of its tibiotarsus suggested it may have been flightless [8]. An additional 19 skeletal elements representing at least two individuals were subsequently attributed to this species [13], but are described and analysed in detail for the first time in the present study. More giant marabou stork remains from Liang Bua have now been identified, almost doubling the available hypodigm, and these include more complete elements from the wings and legs. This increased sample enables a closer examination of the comparative morphology and paleobiology of this extinct species, particularly in terms of its body size, flight capabilities (or lack thereof) and evolutionary history (figure 1). Such details are critical for accurately reconstructing the paleoecology of Pleistocene Flores. Moreover, they may also have important implications for interpretations about how the ancestors of *L. robustus* reached the island in the first place as well as what evolutionary changes occurred subsequently on the island.

## 2. Material and methods

The giant marabou stork skeletal remains that are the focus of this study were recovered during archaeological excavations at Liang Bua and derive from multiple excavated areas called ‘Sectors’ (figure 1 and table 1). Sectors III and IV are each 3 × 3 m areas located roughly in the middle of the cave whereas Sectors VII, XI, XXI, XXII and XXIII are each 2 × 2 m and located near the eastern wall (figure 1). Excavations proceeded in 10 cm intervals (referred to as spits) while following stratigraphic units and the baulks were shored with timber after approximately 2.5 m depth for safety. All items recovered during excavation (e.g. bone, artefact, charcoal) were manually plotted in three dimensions and the sediments from each excavated interval were sieved by hand, followed by wet sieving using 2 mm mesh. Recovered findings were transported to Pusat Penelitian Arkeologi Nasional (Jakarta, Indonesia) for cleaning, cataloging and curation for further study. Avian specimens have a provisional registration number denoted as ‘LB-Av-XX’, where ‘LB’ refers to Liang Bua, and ‘Av’ to Aves.

**Table 1.** Material of *Leptoptilos robustus* from the Late Pleistocene of Liang Bua. Unit refers to the stratigraphic units defined by Sutikna *et al*. [17]. Descriptions of *L. robustus* material and comparisons with extant taxa.

element	ID	sector	spit	unit	side	references
premaxillary	LB-Av-2	XI	46	1B		Meijer *et al*. [13]
mandibula	LB-Av-3072	XV	45	1B		
	LB-Av-3073	XV	45	1B		
cranium	LB-Av-2154/2155	VII	58			
furcula	LB-Av-139	XI	44	1B		Meijer *et al*. [13]
	LB-Av-190	XI	50	1B		Meijer *et al*. [13]
scapula	LB-Av-145	XI	47	1B	R	Meijer *et al*. [13]
	LB-Av-126	XI	50	1B	L	Meijer *et al*. [13]
	LB-Av-2452	XXII	51	1B	R	
	LB-Av-2812	XXI	50	1B	L	
coracoid	LB-Av-2474	XXII	48	1B	R	
	LB-Av-2478	XXII	47	1B	R	
	LB-Av-2740	XXIII	215–265 cm	L		
humerus	LB-Av-107	XI	43	1B		Meijer *et al*. [13]
	LB-Av-179	VII	63	1B	R	
	LB-Av-2470	XXII	48	1B	R	
ulna	LB-Av-134	XI	46	1B		Meijer *et al*. [13]
	LB-Av-135	XI	48	1B		Meijer *et al*. [13]
	LB-Av-148	XI	52	1B		Meijer *et al*. [13]
	LB-Av-154	XI	43	1B	L	Meijer and Awe Due [8]; Meijer *et al*. [13]
	LB-Av-156	XI	47	1B		Meijer *et al*. [13]
	LB-Av-1309	XI	49	1B	R	
	LB-Av-2477	XXII	45	1B	R	
	LB-Av-3283	III	46		R	
radius	LB-Av-115	XI	45	1B	R	Meijer *et al*. [13]
	LB-Av-2300	XXII	47	1B	L	
os carpi radiale	LB-Av-105	XI	45	1B	R	Meijer *et al*. [13]
	LB-Av-106	XI	46	1B	R	Meijer *et al*. [13]
carpometacarpus	LB-Av-1	XI	46	1B	L	Meijer and Awe Due [8]
femur	LB-Av-140	XI	44	1B	L	Meijer and Awe Due [8]
	LB-Av-149 (fr)	XI	46	1B	R	Meijer *et al*. [13]
	LB-Av-2439	XXII	48	1B	R	
tibiotarsus	LB-Av-155	XI	50	1B	L	Meijer and Awe Due [8]
	LB-Av-3360	IV	47	1B	L	
tarsometatarsus	LB-Av-2451	XXII	49	1B	L	
	LB-Av-2476	XXII	42	1B	R	
	LB-Av-2479	XXI	27	3	L	
phalanges	LB-Av-164	I	17	1B	R	
	LB-Av-141	XI	44	1B		Meijer *et al*. [13]
	LB-Av-142	XI	36	1B		Meijer *et al*. [13]
	LB-Av-181	XI	46	1B		Meijer *et al*. [13]
	LB-Av-185	XI	47	1B		Meijer *et al*. [13]

In total, there are currently 43 giant marabou stork elements identified in the Liang Bua faunal assemblage, 20 of which are described here for the first time (table 1). All but four of these elements were recovered directly from stratigraphic Unit 1B, which occurs several stratigraphic units beneath the oldest radiocarbon dated charcoal at the site—OxA-X-2648-13 from Unit 4 yielded a ^14^C age of approximately 46 000 calibrated years before present (ka cal. BP) (95% confidence interval of 47.7–44.1 ka cal.BP) [17,18]—and is considered to be between 120 000 and 60 000 calendar years before present (ka) based on a combination of infrared stimulated luminescence, thermoluminescence, ^234^U/^230^Th and ^40^Ar/^39^Ar dating methods [17]. Two elements (LB-Av-2479 and -2740) were recovered within tephra T3 (Unit 3), a pyroclastic mass flow, and were possibly reworked from the surface of Unit 2 (approx. 60–50 ka) or perhaps Unit 1B (approx. 120–60 ka) when T3 was initially deposited [17,18]. Another two elements (LB-Av-2154 and -2155) were recovered from Unit 6, a younger stratigraphic unit; however, these were both most likely reworked from Unit 1B because in this particular area of the cave, these younger units occur downslope and unconformably overlie Unit 1B [17,18].

Skeletal elements from 38 extant specimens of *Ephippiorhynchus asiaticus, E. senegalensis, Leptoptilos crumenifer, L. dubius* and *L. javanicus* were measured for comparisons with the Liang Bua material. These 38 specimens are curated at the following institutions: Smithsonian Institution's National Museum of Natural History in Washington DC, USA (prefix USNM), Senckenberg Natural History Museum in Frankfurt, Germany (SMF), the Natural History Museum in London, UK (prefix NHMUK), the Royal Belgian Institute of Natural Sciences in Brussels, Belgium (prefix RBINS), Natural History Museum of Denmark in Copenhagen (prefix KUZM) and the University Museum of Bergen, Norway (prefix BM). Further details about these comparative specimens are provided in electronic supplementary material, table S1.

Osteological terminology primarily follows Baumel & Witmer [20] and Howard [21]. Measurements were taken using digital calipers to 0.01 mm. In select cases where measurements were taken from digital images, ImageJ was used [22]. For the Liang Bua giant marabou stork bones, all surfaces were examined for evidence of postmortem modifications (e.g. digestion marks (following [23]), gnawing marks, cut marks, etc.) using a handheld 10× lens. Bone weathering stage (BWS) was scored according to Behrensmeyer [24]. The porosity and texture of the bone surfaces were used to distinguish juveniles from adults.

## 3. Results

### 3.1. Systematic palaeontology

Class Aves [25]

Order Ciconiiformes [26]

Family Ciconiidae [27]

Tribe Leptoptilini [28]

Genus *Leptoptilos* [29]

*Leptoptilos robustus* [8]

### 3.2. Emended diagnosis

Body size larger than *L. dubius* and *L. crumenifer* although smaller *L. robustus* individuals overlap in size with *L. dubius* and larger *L. crumenifer.* Morphology and intra- and inter-limb proportions similar to those in extant *Leptoptilos.* Morphology and intra-limb proportions also similar to those of *L. falconeri.* However, *L. robustus* differs from *L. falconeri* in having similar hind-to-forelimb proportions as in extant *Leptoptilos*.

### 3.3. Newly referred material (table 1)

Mandibular fragments LB-Av-3072/3073; cranial fragments LB-Av-2154/2155; right scapula LB-Av-2452; left scapula LB-Av-2812; right coracoid LB-Av-2478; right coracoid LB-Av-2474; left coracoid LB-Av-2740; distal right humerus LB-Av-2470; distal right humerus LB-Av-179; right ulna LB-Av-3283; proximal right ulna LB-Av-1309; proximal right ulna LB-Av-2477; right proximal radius LB-Av-115; left proximal radius LB-Av-2300; right femur LB-Av-2439; distal left tibiotarsus LB-Av-3360; right tarsometatarsus LB-Av-2476; left tarsometatarsus LB-Av-2451; left tarsometatarsus shaft LB-Av-2479; right pedal phalanx 1 LB-Av-164. Measurements for the coracoid, scapula, humerus, ulna, femur, tibiotarsus, and tarsometatarsus for *L. robustus* and extant *Leptoptilos* and *Ephippiorhynchus* species are given in tables 2–9.

**Table 2.** Mean, minimum and maximum measurements (in mm) of the scapula of *Leptoptilos robustus* and extant species of *Leptoptilos* and *Ephippiorhynchus.* GL, greatest length of specimen, LAH, length from the tip of the acromion to the distal edge of the humeral facet.

taxon		GL	LAH
*L. robustus*	LB-Av-126		35.4
*L. robustus*	LB-Av-145		35.3
*L. robustus*	LB-Av-2452		37
*L. crumenifer* (*n* = 14)		125.6 (11.7–138.9)	30.9 (28–34.2)
*L. javanicus* (*n* = 9)		103.1 (97.8–110.1)	24.9 (22.4–27.6)
*L. dubius* (*n* = 11)		138.1 (122–145)	35.2 (31.2–39)
*E. asiaticus* (*n* = 3)		99.6 (95–107)	22.6 (22.5–22.8)

**Table 3.** Mean, minimum and maximum measurements (in mm) of the coracoid of *Leptoptilos robustus* and extant species of *Leptoptilos* and *Ephippiorhynchus* (1 *E. senegalensis,* 3 *E. asiaticus*)*.* GL, greatest length of specimen; WAP, width across the acrocoracoid process; LHA, length from distal rim of the humeral facet to the dorsal tip of the acrocoracoid process; LHF, length of the humeral facet.

taxon		GL	WAP	LHA	LHF
*L. robustus*	LB-Av-2474		35.8	51	28.3
*L. robustus*	LB-Av-2478		36.4	50.8	31.2
*L. crumenifer* (*n* = 14)		129.6 (116–147.1)	33.9 (30.3–38.9)	46.4 (40.7–51.8)	26.1 (23.4–28.1)
*L. javanicus* (*n* = 9)		101.3 (96.6–108.2)	25.7 (23.5–28.1)	36.4 (33.3–39.7)	18.2 (13–22.5)
*L. dubius* (*n* = 11)		142.5 (128.4–151)	38.8 (34.4–42.5)	52.7 (47.2–56.8)	28.9 (25.5–31)
*Ephippiorhynchus* (*n* = 4)		96.3 (88.3–107.7)	21.2 (18–24.9)	33.7 (31–39.8)	17.9 (16.8–18.9)

**Table 4.** Mean, minimum and maximum measurements (in mm) of the humerus of *Leptoptilos* *robustus* and extinct and extant species of *Leptoptilos* and *Ephippiorhynchus* (1 *E. senegalensis,* 3 *E. asiaticus*). DW, distal width, DD, distal depth. Size data for *L. falconeri* from Harrison [30], for *L. lüi* from Zhang *et al*. [2].

taxon		DW	DD
*L. robustus*	LB-Av-179	53.2	
*L. robustus*	LB-Av-2470	55.9	
*L. crumenifer* (*n* = 14)		46.9 (43–51.9)	24.6 (22.7–27.2)
*L. javanicus* (*n* = 9)		37 (34.2–40.3)	20.3 (19–21.4)
*L. dubius* (*n* = 11)		52.8 (46.3–57.8)	27.3 (24–29.6)
*Ephippiorhynchus* (*n* = 4)		35.3 (32–40)	18.1 (17–20)
*L. falconeri*	NHMUK PV OR 48435	57.7	
*L. lüi*	SAM 94. J. VIII-13.C-11	56.9

**Table 5.** Mean, minimum and maximum measurements (in mm) of the ulna of *Leptoptilos robustus* and extinct and extant species of *Leptoptilos* and *Ephippiorhynchus* (1 *E. senegalensis,* 3 *E. asiaticus*). GL, greatest length of the specimen, PW, proximal width, PD, proximal depth, DW, distal width, DD, distal depth, MWS, minimal width of the shaft, MDS, minimal depth of the shaft. Measurement for *L.* cf. *falconeri* taken from an image.

taxon		GL	PW	PD	DW	DD	MWS	MDS
*L. robustus*	LB-Av-154				25.9	21.9	14.7	15.3
*L. robustus*	LB-Av-156						16.1	14.6
*L. robustus*	LB-Av-2477		36.4	28.6			16.6	14.9
*L. robustus*	LB-Av-3283				29.7	24.8	19.1	18.3
*L. crumenifer* (*n* = 14)		370.3 (329–419)	31.5 (28.5–34.2)	25.9 (23.4–27.3)	23.6 (21.5–26.4)	19.5 (17–22)	13.3 (11.9–15.3)	12 (10.5–13.8)
*L. javanicus* (*n* = 9)		285.1 (261.5–307)	24.6 (23–27.3)	21.2 (20.3–22.8)	18.7 (17.3–20.3)	16.6 (15.1–17.6)	10.9 (10.2–12)	10.2 (9.5–11)
*L. dubius* (*n* = 10)		416.3 (363–435)	34.9 (31.2–36.8)	28.0 (26–29.9)	25.8 (22.5–28)	21.4 (19.2–23.6)	14.6 (13.5–15.7)	13 (11.8–14)
*Ephippiorhynchus* (*n* = 4)		277.2 (254–310)	23.6 (21.3–26.3)	18.9 (17.4–21)	17.5 (15.6–19.7)	14.3 (13.1–16)	10.4 (9.3–12)	8.1 (7.3–9)
*L.* cf*. falconeri*	KNM-KP 50764				∼28			
*L.* cf*. dubius*	RGM.DUB.1491						15	13.2
*E.* cf*. asiaticus*	RGM.DUB.5913		22.6	19.7

**Table 6.** Mean, minimum and maximum measurements (in mm) of the carpometacarpus of *Leptoptilos robustus* and extant species of *Leptoptilos* and *Ephippiorhynchus* (1 *E. senegalensis,* 3 *E. asiaticus*)*.* GL, greatest length of the specimen, PW, proximal width, PD, proximal depth.

taxon		GL	PW	PD
*L. robustus*	LB-Av-1		24.6	18.3
*L. crumenifer* (*n* = 14)		160.6 (144–183.9)	32.8 (30.1–35.9)	14.9 (13.3–16.8)
*L. javanicus* (*n* = 9)		128.2 (118–137.5)	26.6 (24.8–28.4)	12.1 (11.3–13.5)
*L. dubius* (*n* = 9)		174.6 (155–185)	35.0 (30–37.9)	16.3 (14.9–17.3)
*Ephippiorhynchus* (*n* = 4)		117.9 (108.4–129)	23.6 (22–27.5)	10.9 (9.6–12)

**Table 7.** Mean, minimum and maximum measurements (in mm) of the femur of *Leptoptilos robustus,* fossil *Leptoptilos,* and extant species of *Leptoptilos* and *Ephippiorhynchus* (1 *E. senegalensis,* 3 *E. asiaticus*)*.* GL, greatest length of the specimen, PW, proximal width, PD, proximal depth, DW, distal width, DD, distal depth, MWS, minimal width of the shaft, MDS, minimal depth of the shaft. Measurement for *L. falconeri* taken from image.

taxon		GL	PW	PD	DW	DD	MWS	MDS
*L. robustus*	LB-Av-140	139	31	21.3	35.8	32.7	21	18.9
*L. robustus*	LB-Av-2439	144	40		38	30.8	18.7	17.9
*L. crumenifer* (*n* = 14)		130.1 (117–144.6)	34.4 (31–38.4)	19.6 (17.9–21.4)	33 (29.5–35.5)	29.8 (26–33.7)	17.1 (14.8–19.3)	15.9 (14.1–17.6)
*L. javanicus* (*n* = 9)		110.5 (103.2–116.4)	27 (22.2–30)	16.8 (15.4–18.7)	27.4 (25.6–29.4)	25.5 (23.6–27.3)	13.8 (12.5–15.3)	13.1 (12–14.1)
*L. dubius* (*n* = 11)		142.7 (126.9–157)	38.8 (33.4–42.8)	22.8 (19.3–24.4)	36.9 (32.9–40.9)	32.2 (28.4–36)	19.6 (17.1–21.8)	18 (15.8–20.6)
*Ephippiorhynchus* (*n* = 4)		116.1 (109.3–126.8)	28 (25.2–32)	17.3 (15.6–19)	27.1 (24.9–29.7)	25.7 (24–29.5)	13.3 (12.3–14.7)	12.8 (11.4–15)
*L. falconeri*	NHMUK PV OR 39737				∼47

**Table 8.** Mean, minimum and maximum measurements (in mm) of the tibiotarsus of *Leptoptilos robustus,* fossil *Leptoptilos,* and extant species of *Leptoptilos* and *Ephippiorhynchus* (1 *E. senegalensis,* 3 *E. asiaticus*)*.* GL, greatest length of the specimen, DW, distal width, DD, distal depth, MWS, minimal width of the shaft, MDS, minimal depth of the shaft. Measurements for *L. falconeri, L.* cf. *falconeri, L. dubius/falconeri* and Leptoptilini gen. et sp. indet from Louchart *et al*. [1].

taxon		GL	DW	DD	MWS	MDS
*L. robustus*	LB-Av-155				14.8	13.4
*L. robustus*	LB-Av-3360				12.9	12.3
*L. crumenifer* (*n* = 14)		360.4 (320–420)	20.7 (19.1–22.6)	26.1 (23.8–28.3)	11.7 (10.8–13.4)	10.5 (9.5–11.4)
*L. javanicus* (*n* = 9)		306.8 (282–351)	17 (15.8–17.8)	21.9 (20.5–24.1)	10.1 (9.4–11.1)	8.9 (8.5–9.7)
*L. dubius* (*n* = 10)		391.4 (352–422)	22 (19.7–24.5)	29.6 (26.2–32.2)	12.3 (11.1–13.2)	11.6 (10.1–12.5)
*Ephippiorhynchus* (*n* = 4)		366.1 (327.3–415)	17.4 (15.7–20)	23.9 (21.6–27.2)	9.4 (8.3–10.7)	9 (8.1–9.9)
*L*. cf. *dubius*	RGM.DUB.1490				11.9	10.9
*L. falconeri*	KB3.97.161				17.5	16
*L. falconeri*	OMO-122-76-367		27.4	34.2	17.2	
*L. falconeri*	SAG-VP-1/19				14.8	14.1
cf. *L. falconeri*	URU-VP-1/28		26	34.5		
cf. *L. falconeri*	URU-VP-1/15			35		
*L. dubius*/*falconeri*	NHMUK PV OR 48444		22	25.6		
Leptoptilini gen. et sp. Indet.	NHMUK PV OR 39734		24.4	31.6	15.3	13.8

**Table 9.** Mean, minimum and maximum measurements (in mm) of the tarsometatarsus of *Leptoptilos robustus*, fossil *Leptoptilos* and extant species of *Leptoptilos* and *Ephippiorhynchus* (1 *E. senegalensis,* 3 *E. asiaticus*). GL, greatest length of the specimen, PW, proximal width, PD, proximal depth, DW, distal width, DD, distal depth, MWS, minimal width of the shaft, MDS, minimal depth of the shaft. Measurements for *L. falconeri* and *L*. cf. *falconeri* from [1].

taxon		GL	PW	PD	DW	DD	MWS	MDS
*L. robustus*	LB-Av-2451						12.1	9.6
*L. robustus*	LB-Av-2476		31.6	17.5			14.1	14.6
*L. robustus*	LB-Av-2479	293	27.6	17	34.4	21.6	12.4	9
*L. crumenifer* (*n* = 14)		283.2 (244–327)	24.2 (21.8–26.3)	15.1 (12.5–17)	28.9 (25.4–30.7)	20.0 (18.1–21.9)	10.7 (9.4–11.8)	7.6 (6.4–8.3)
*L. javanicus* (*n* = 9)		236.1 (213.2–262.5)	20.4 (18.8–21.5)	12.9 (12–14.3)	24.6 (23.2–26)	16.8 (15.1–17.5)	9.1 (8.5–9.9)	6.1 (5.5–6.4)
*L. dubius* (*n* = 10)		313.6 (277.5–345)	26.9 (23.6–29.3)	16.7 (14.6–18.3)	30.6 (27–32.4)	21.7 (18.7–23.6)	11.3 (10.6–12)	8.3 (7.3–9.5)
*Ephippiorhynchus* (*n* = 4)		322.8 (293.3–355)	21.1 (19.4–22.8)	13.0 (11.2–14.7)	23 (21.5–25.1)	17.3 (16.1–18.5)	8.1 (7.3–9)	6.4 (5.5–7.2)
*L. titan*	GSI 3313	372.9	31.5	16.8			12.4	9.9
*L. falconeri*	NHMUK PV OR 39736				36.0			
*L. falconeri*	SAG-VP-1/19	405			36.5	25.6	13.2	
*L.* cf. *falconeri*	IZAN 8024	315			32.2	21	13.2	

#### 3.3.1. Skull

**Figure 2.**
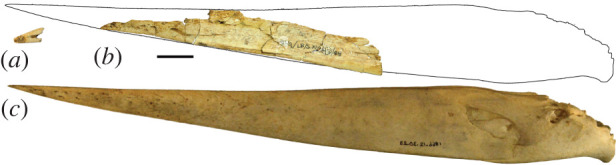
Cranial elements: (*a*), maxillary fragment (LB-Av-2) of *L. robustus*; (*b*), mandibular fragment (LB-Av-3072/73) of *L. robustus* and (*c*), mandible (mirror image) of *L. crumenifer* (NMHUK 1866.12.30.23).

Preserved elements of the skull include a tip of a maxilla (LB-Av-2; figure 2*a*), several fragments that form part of a left mandibular ramus (LB-Av-3072/3073; figure 2*b*) and two undiagnostic fragments from the paroccipital region of the cranium (LB-Av-2154/2155). Like in extant *Leptoptilos*, the maxillary tip displays a flat rostrum maxillare and its cristae tomiales are not distinct, while its most proximal foramen neurovasculare terminates in a small sulcus rostrally [13]. Although fragmentary, the mandibular ramus is dorsoventrally high as is typical for Ciconiidae (figure 2*c*). Several foramina neurovascularia are visible near the crista tomialis but in addition, the overall surface of the ramus fragment shows elongated pores indicative of juvenile bone. It is unclear if the mandibular ramus fragments belong to the same individual as the maxillary tip, but both come from the same layer and similar depth of adjacent sectors.

#### 3.3.2. Furcula

**Figure 3.**
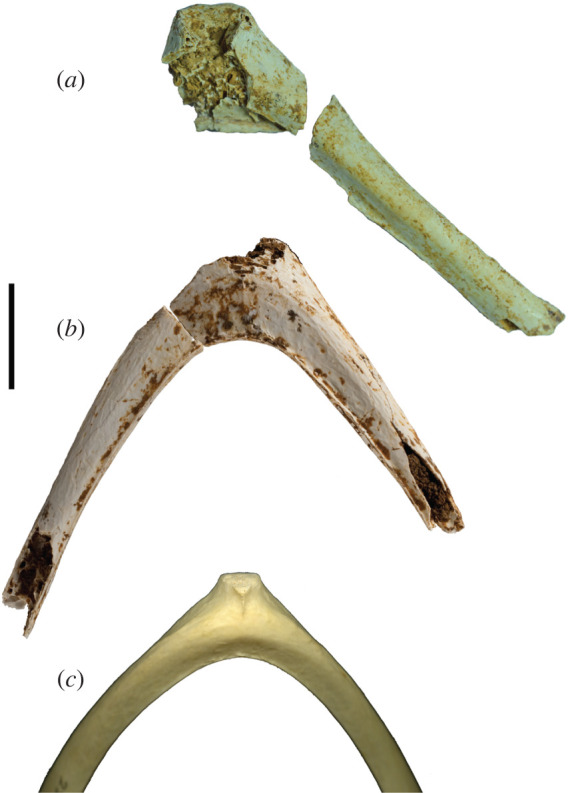
Furculae (in cranial view): (*a*), partial furcula of *L. robustus* (LB-Av-139); (*b*), furcula of *L. robustus* (LB-Av-190) and (*c*), furcula of *L. dubius* (NMNH 225988).

Two furculae (LB-Av-190 and -139) represent two individuals. LB-Av-139 (figure 3*a*) is larger than LB-Av-190 (figure 3*b*) and both are larger than in *Leptoptilos crumenifer* (figure 3*c*). They display a sharp, ridge-like dorsal edge of the clavicula as well as wide and flat apophysis furculae [13]. Like in extant *Leptoptilos*, the synostosis interclavicularis is short and blunt rather than elongated and ventrally projected as it is in *Trigonoceps*, and the angle at which the claviculae join at the apophysis is approximately 75° [13].

#### 3.3.3. Scapula

**Figure 4.**
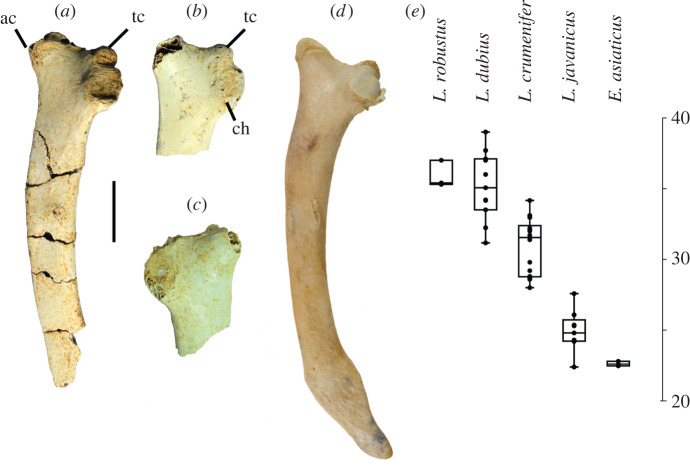
Scapulae (in lateral view): (*a*), right scapula of *L. robustus* (LB-Av-2452); (*b*), proximal right scapula of *L. robustus* (LB-Av-145); (*c*), proximal left scapula of *L. robustus* (LB-Av-126); (*d*), right scapula of *L. crumenifer* (NMHUK 1866.12.30.23); (*e*) box plots of the length from the acromion to the scapular facet in the scapula of extinct and extant species of *Leptoptilos* and *Ephippiorhynchus*. ac, acromion; tbc, tuberculum coracoideum.

Four fragmentary scapulae (LB-Av-126, -145, -2452, -2812) represent at least two individuals. LB-Av-2812 is a proximal left scapula that has been crushed and consists of fragments only. LB-Av-2452 (figure 4*a*) is from the right side and lacks the distal end. LB-Av-145 (figure 4*b*) and -126 (figure 4*c*) are right and left proximal ends, respectively. The latter three bones display a prominent rounded tuberculum coracoideum that projects cranially beyond the facies articularis humeralis, and an acromion that is wide with a blunt top and sits at a 30° angle to the long axis of the shaft. This morphology agrees with that of *Leptoptilos* (figure 4*d*)*,* although the tuberculum coracoideum appears less prominent in *Leptoptilos javanicus.* The length from the tip of the acromion to the distal edge of the humeral facet in LB-Av-2452, -145 and -126 overlaps with the range of values for *L. dubius* (table 2 and figure 4*e*).

#### 3.3.4. Coracoid

**Figure 5.**
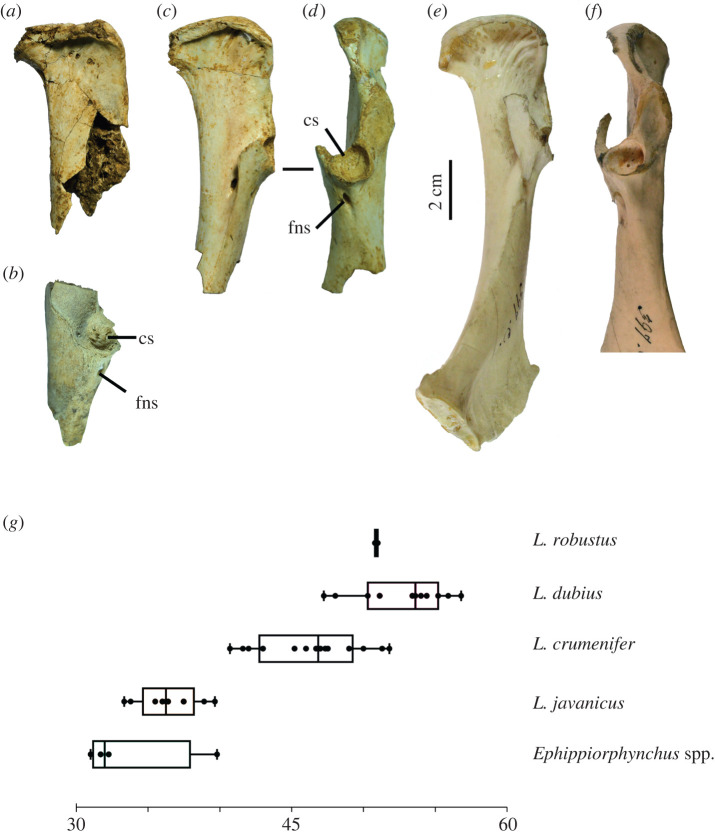
Coracoids (in medial (*a*, *c*, *e*) and dorsal view (*b*, *d*, *f*)): (*a*), right coracoid of *L. robustus* (LB-Av-2478); (*b*), left coracoid of *L. robustus* (LB-Av-2740); (*c-d*), right coracoid of *L. robustus* (LB-Av-2474); (*e-f*), Right coracoid of *L. crumenifer* (NMHUK 1866.12.30.23). (Image courtesy of NHM); (*g*), box plots of the width of the acrocoracoid of extinct and extant species of *Leptoptilos* and *Ephippiorhynchus*. cs, cotyla scapularis; fns, foramen nervi supracoracoidei.

Three partial coracoids (LB-Av-2740, -2474, and -2478) represent at least two individuals (table 3 and figure 5*a–d*). LB-Av-2478 and -2474 are both from the right side and preserve the proximal articulation, which is broad and flat (figure 5*a,c*). The processus acrocoracoideus ventrally overhangs the ventral margin of the bone, similar to *Leptoptilos* but unlike other Ciconiidae [31]. The length and width of the processus acrocoracoideus in LB-Av-2478 and -2474 fall within the size ranges of both *L. dubius* and *L. crumenifer* (table 3)*.* In LB-Av-2478, but not in -2740, a foramen is visible on the ventral margin of the bone, distal to the processus acrocoracoideus, the facies articularis clavicularis is wide but flat and only minimally developed, and the sulcus m. supracoracoidei is broad and contains several foramina. The cotyla scapularis of LB-Av-2478 and -2740 is round and deep. A foramen nervi supracoracoidei is present in the processus procoracoideus (figure 5*b*,*c*), a feature that within extant Ciconiidae is only present in *Leptoptilos* [31]*.* A pneumatic foramen is present at the same level on the corpus coracoidei.

#### 3.3.5. Humerus

**Figure 6.**
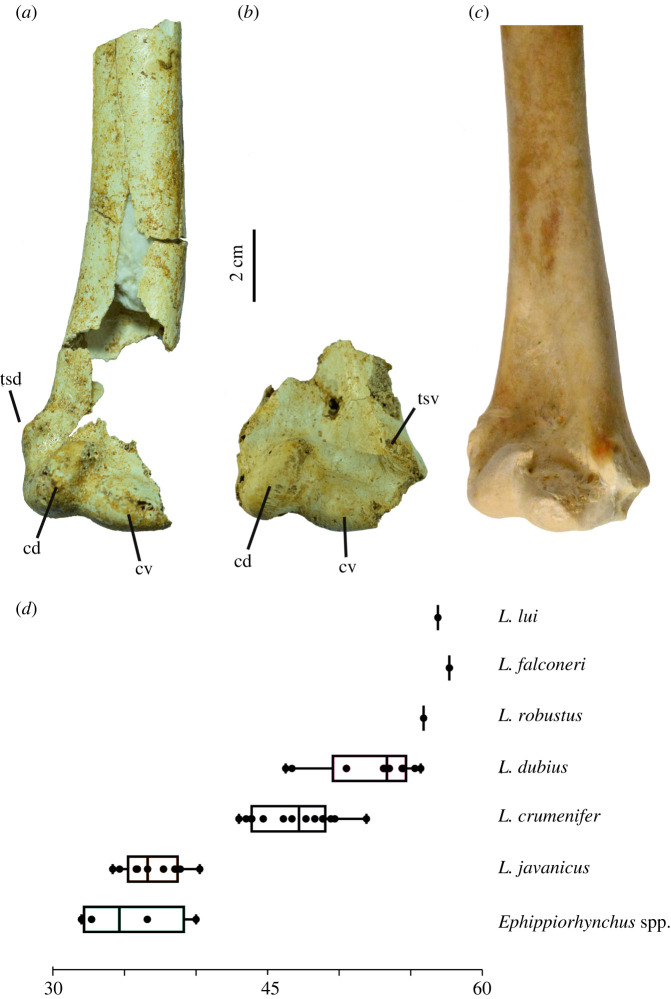
Humeri (in palmar view): (*a*), right distal humerus of *L. robustus* (LB-Av-179); (*b*), right distal humerus of *L. robustus* (LB-Av-2470); (*c*), right distal humerus of *L. crumenifer* (NMHUK 2014.65.1) (Image courtesy of NHM); (*d*), box plots of humerus distal widths of extinct and extant species of *Leptoptilos* and *Ephippiorhynchus.* cd, condylaris dorsalis; cv, condylaris ventralis; tsd, tuberculum supracondylare dorsale; tsv, tuberculum supracondylare ventrale.

Two distal right humeri (LB-Av-179 and -2470) and an isolated caput humeri (LB-Av-107) are preserved (figure 6*a,b*). LB-Av-107 preserves no diagnostic features but its dimensions (37.6 mm in width and 18.9 mm in depth) agree with *Leptoptilos*. LB-Av-2470 consists of only the distal end whereas the distal shaft of LB-Av-179 is partially preserved, but the fossa m. brachialis is broken away. LB-Av-2470's fossa m. brachialis is preserved, however, and it is broad, deep and appears to extend proximally beyond the edge of the fragment. Several small pneumatic foramina are visible directly above the incisura intercondylaris, with a larger foramen (likely eroded to a larger size than the original one) located more proximally. The condylus dorsalis is oriented proximo-ventrally and tapers gradually proximally. In both specimens, the very proximal tip of the condylus dorsalis is broken off, but LB-Av-2470's condyles dorsalis curves ventrally suggesting that the proximal tip did so as well, like the condition observed in extant *Leptoptilos*. In both specimens, the condylus ventralis is oriented dorsoventrally, and similar in length to the long axis as the condylus dorsalis. The tuberculum supracondylare dorsale, preserved in LB-Av-179, is well-developed and forms a prominent, low triangle on the shaft of the bone. Although both specimens are damaged, they are similar in size to the largest specimens of *L. dubius* and LB-Av-2470 preserves a transverse width of 50.9, but likely lacks approximately 5 mm due to damage (table 4).

#### 3.3.6. Ulna

**Figure 7.**
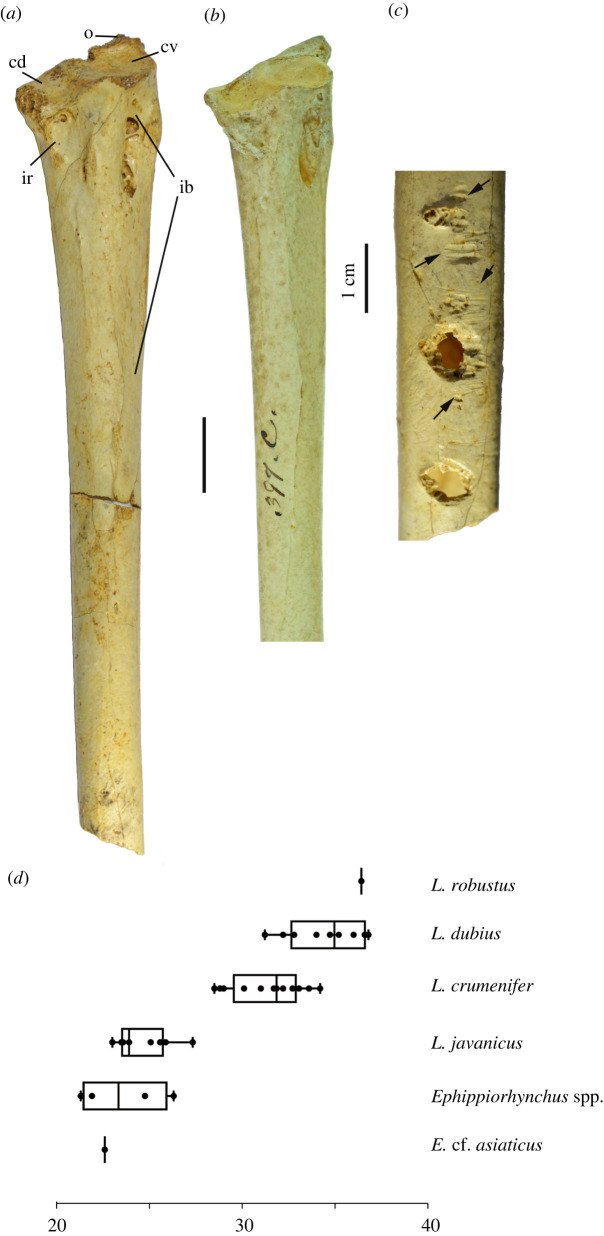
Proximal ulnae (in ventral view): (*a*), proximal right ulna of *L. robustus* (LB-Av-2477); (*b*), proximal right ulna of *L. crumenifer* (S/2014.65.1); (*b*), detail of the dorsal side of LB-Av-2477, arrows indicate rodent gnawing marks; (*d*), box plots of ulnar proximal width of extinct and extant species of *Leptoptilos* and *Ephippiorhynchus*. cd, cotyla dorsalis; cv, cotyla ventralis; ib, impressio m. brachialis; ir, incisura radialis; o, olecranon.

Eight ulnar fragments (LB-Av-134, -135, -148, -154, -156, -1309, -2477, -3283) represent at least three individuals. LB-Av-2477 is the only proximal element of the ulna that is preserved (figure 7*a*). Its proximal width and depth overlap with that of the two largest specimens of *L. dubius* whereas its midshaft dimensions exceed those of the extant taxa (table 5). The tip of the olecranon is missing, as is the processus cotylaris dorsalis. The cotyla ventralis is large and subcircular, while the cotyla dorsalis is a smaller, sloping surface on the dorsal side of the cotyla ventralis. Both the incisura radialis (distal from the cotyla dorsalis) and the impressio m. brachialis are distinct, deep, and display several pneumatic foramina and bony struts, like the condition seen in extant *Leptoptilos.* On the caudal side of the bone, the papillae remigales are damaged and in some cases entirely missing, with small parallel marks varying in widths—characteristic of rat gnawing marks—appearing adjacent to each (figure 7*c*).

**Figure 8.**
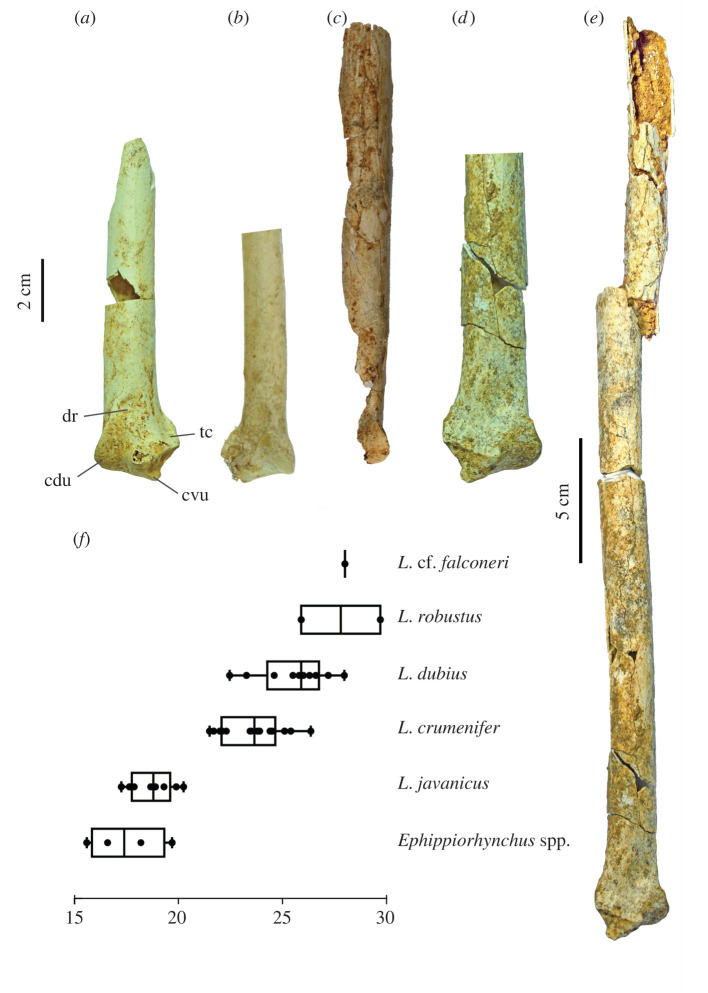
Distal ulnae (in ventral view): (*a*), distal left ulna of *L. robustus* (LB-Av-154); (*b*), distal right ulna of *L. crumenifer* (NHMUK S/2014.65.1) (Image courtesy of NHM); (*c*), distal right ulna of *L. robustus* (LB-Av-1309); (*d-e*), right ulna of *L. robustus* (LB-Av-3283); (*f*), box plots of ulnar distal width or extinct and extant species of *Leptoptilos* and *Ephippiorhynchus.* cdu, condylus dorsalis ulnae; cvu, condylus ventralis ulnae; dr, depressio radialis; tc, tuberculum carpale.

LB-Av-3283 is an almost complete ulna, with only the proximal articulation missing (figure 8*d,e*), and it is considerably larger than ulnae from the extant comparative sample in all measurable dimensions (table 5). Its preserved length is 372 mm, but if complete, it would likely measure at least 2 cm longer. LB-Av-3283 is broken in several places and the bone surface is weathered but the papillae remigales remain visible. It is very similar in morphology to LB-Av-154 (figure 8*a*), which is smaller and overlaps in size with larger *L. crumenifer* and *L. dubius* specimens except for its minimum shaft depth. Distally, the depression radialis is distinct but shallow. The condylus ventralis ulnae is pointed and oriented dorsally. In dorsal view, a distinct foramen occurs between the condylus ventralis ulnae and the tuberculum carpale. This foramen is also present in LB-Av-154 and in all extant and fossil *Leptoptilos* [3]*,* but not *Ephippiorhynchus* [8]*.* Like in LB-Av-154, the condylus dorsalis ulnae of LB-Av-3283 is a flattened ridge that extends onto the shaft. LB-Av-1309 is a partial distal right ulna with only the condylus dorsalis ulnae and a portion of the shaft remaining (figure 8*c*). The condylus dorsalis ulnae displays the flattened ridge seen in the other specimens and although the dimensions of the shaft cannot be assessed, overall, it appears comparable with the other ulnae attributed to *L. robustus.* LB-Av-156 is a shaft fragment with a minimum shaft width and depth that slightly exceeds that of the extant sample.

#### 3.3.7. Radius

**Figure 9.**
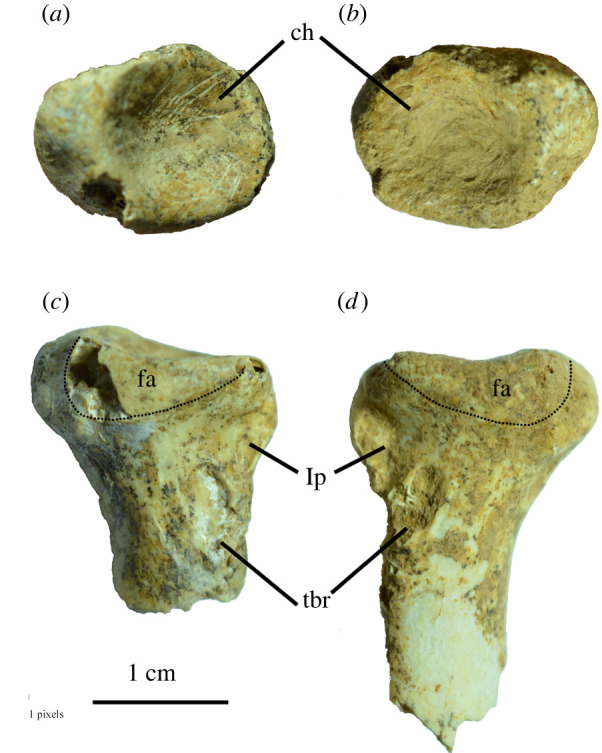
Radii of *L. robustus* ((*a*) and (*b*) in proximal view; (*c*) and (*d*) in lateral view): (*a*), left proximal ulna LB-Av-2300; (*b*), right proximal ulna LB-Av-115; (*c*), left proximal ulna LB-Av-2300; (*d*), right proximal ulna LB-Av-115. fa, facies articularis ulnaris, ch, cotyla humeralis, lp, ligamental papilla, tbr, tuberculum bicipitale radii.

A right and left proximal radius (LB-Av-115 and -2300, figure 9*a,b*) are similar to one another in size of the proximal articulation (18.6 × 13.8 mm and 18.1 mm × 14.3 mm, respectively) and may represent the same individual. Both specimens have a subcircular humeral cotyla, and the ‘lip’-like facies articularis ulnaris sits on its lateral side. Distal to the humeral cotylar rim on the palmar surface of the shaft sits a thin ridge that seems pinched medio-laterally, which is the ligamental papilla that forms the attachment for the cranial cubital ligament. The tuberculum bicipitale radii carries distinct notches on its medial and lateral side for the attachment of the m. biceps brachii occurs distally from the papilla.

#### 3.3.8. Carpometacarpus

A partial left carpometacarpus (LB-Av-1) consists of the proximal half of the os metacarpale majus and the trochlea carpalis. The os metacarpale alulare, processes extensorius and processus pisiformis are not preserved. Similar to extant *Leptoptilos*, it displays a pneumatic foramen in the fossa infratrochlearis. The trochlea carpalis is proximally very shallow but deepens distally and ends in a distinct fovea carpalis caudalis. It is unclear if there is a pneumatic foramen in the fovea carpalis caudalis because sediment obscures the bone surface. It has a proximal end depth that is larger than in extant *Leptoptilos* (table 6) [8].

#### 3.3.9. Femur

**Figure 10.**
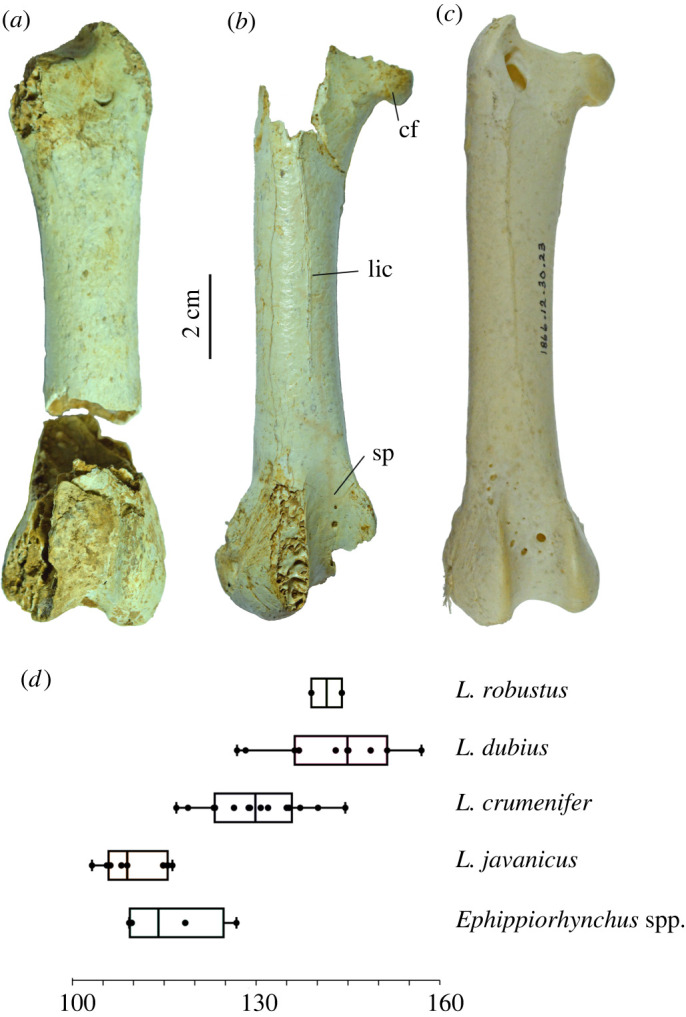
Femora (in cranial view): (*a*), left femur of *L. robustus* (LB-Av-140); (*b*), right femur of *L. robustus* (LB-Av-2439); (*c*), right femur of *L. crumenifer* (NHMUK 1866.12.30.23) (Image courtesy of NHM); (*d*), box plots of femur length for extinct and extant species of *Leptoptilos* and *Ephippiorhynchus*.

Three femoral fragments (LB-Av-140, -149 and -2439) represent three individuals (figure 10*a,b*). LB-Av-140 and -2439 are from the left and right sides, respectively, but their dimensions differ from one another enough that it is unlikely that they are from the same individual, and -149 consists of right shaft and distal articulation fragments that represent a second right femur (table 7). The distal widths of LB-Av-140 and -2439 fall within the size range of *L. dubius* although the former is also comparable to the largest *L. crumenifer* (table 7). LB-Av-149 consists of fragments of the lateral condyle and the fossa poplitea and matches the other two specimens in morphology and general size, but no other diagnostic features are preserved. LB-Av-2439 lacks the trochanter femoris but partially preserves the caput femoris. The linea intermuscularis cranialis is distinct and attains a more central position on the cranial surface than in LB-Av-140, like in extant *Leptoptilos.* Distally, the sulcus patellaris is broad and contains several pneumatic foramina, which are also observed in extant *Leptoptilos* (figure 10*c*)*.* The lateral condyle is broken off, but the medial condyle is robust with a rugose medial surface. The epicondylus lateralis sits atop the ridge-like proximal edge of the medial surface. Distally, there is a distinct impressio ligamentum collateralis lateralis. Caudally, the fossa poplitea is deep, similar to that in LB-Av-140.

#### 3.3.10. Tibiotarsus

**Figure 11.**
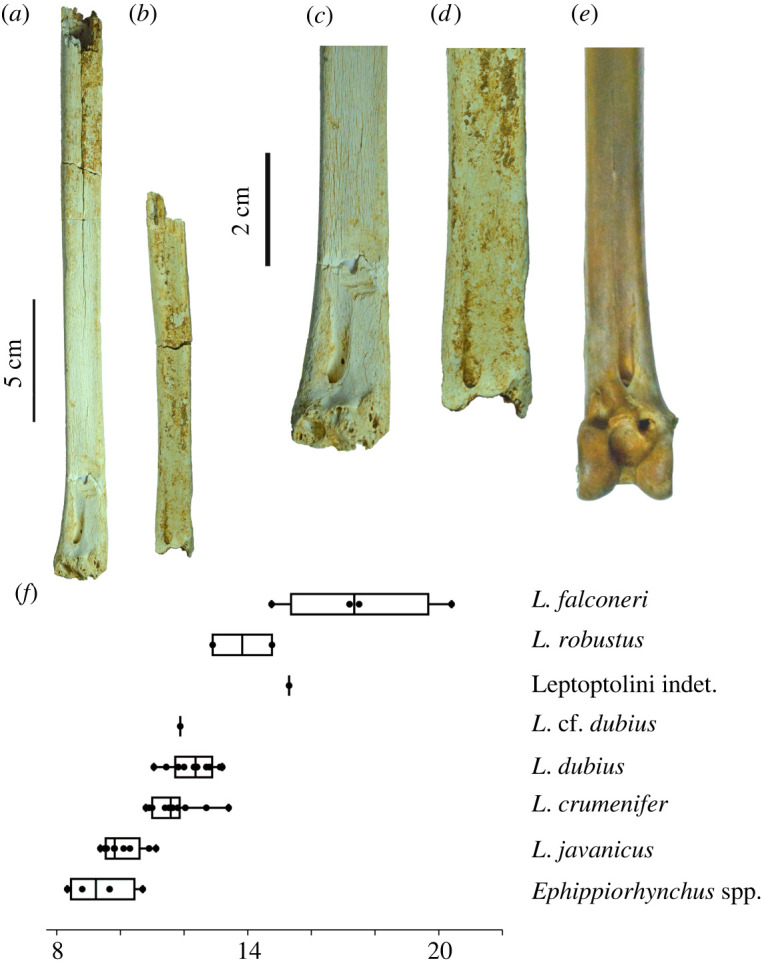
Tibiotarsi (in cranial view): (*a*), distal left tibiotarsus of *L. robustus* (LB-Av-155); (*b*), distal left tibiotarsus of *L. robustus* (LB-Av-3360); (*c*), close-up of distal left tibiotarsus of *L. robustus* (LB-Av-155); (*d*), close-up of distal left tibiotarsus of *L. robustus* (LB-Av-3360); (*e*), distal right tibiotarsus of *L. crumenifer* (NHMUK 1866.12.30.23) (Image courtesy of NHM); (*f*), box plots of tibiotarsus shaft width for extinct and extant species of *Leptoptilos* and *Ephippiorhynchus*.

Two left tibiotarsi (LB-Av-155 and -3360) (figure 11*a–d*) represent two individuals. LB-Av-155 is the holotype [8] and there are small longitudinal pores visible on the bone surface suggesting that it was juvenile. LB-Av-3360 consists of the distal shaft and lacks the distal end below the level of the pons supratendineus, which is incomplete and precludes an assessment of the position of its distal opening. At the proximal end of the fragment, the cortical bone wall measures 2.5–2.8 mm. The sulcus extensorius appears slightly shorter and in a more medial position on the shaft than in LB-Av-155. On the lateral and caudal surface of the distal end, parallel rat gnawing marks are present. The minimum shaft width and depth of LB-Av-3360 overlaps with the largest *L. dubius* and *L. crumenifer* specimens*,* whereas the minimum shaft dimensions of LB-Av-155 exceed those for extant *Leptoptilos* (table 8 and figure 11*d*).

#### 3.3.11. Tarsometatarsus

**Figure 12.**
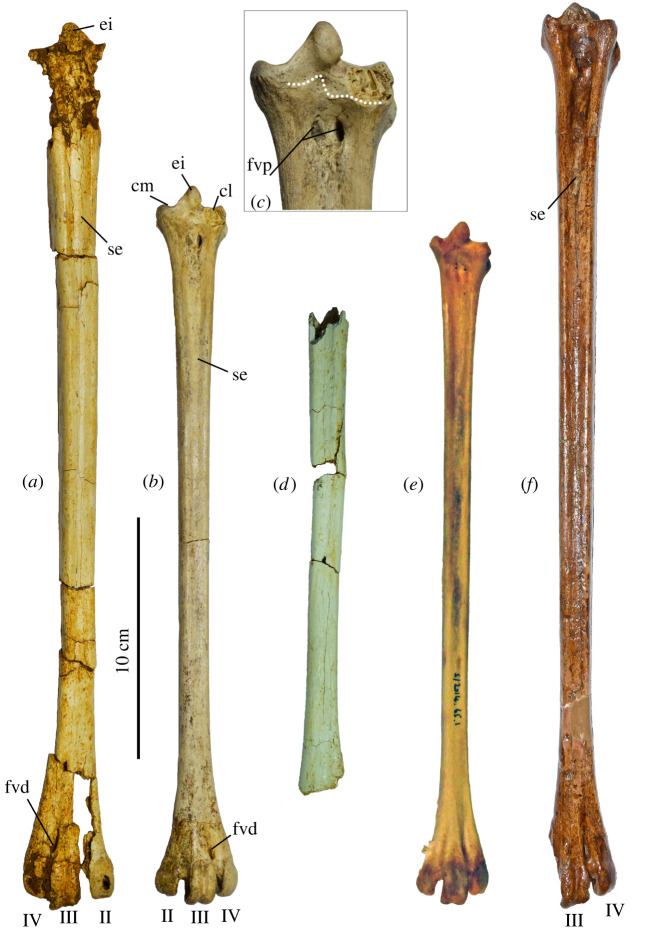
Tarsometatarsi (in cranial view): (*a*), right tarsometatarsus of *L. robustus* (LB-Av-2476); (*b*), left tarsometatarsus of *L. robustus* (LB-Av-2451); (*c*), detail of proximal articulation of LB-Av-2451. Dotted line indicates suture; (*d*), shaft of left tarsometatarsus of *L. robustus* (LB-Av-2479); (*e*), left tarsometatarsus of *L. crumenifer* (NHMUK S2014.65.1) (Image courtesy of NHM); (*f*), left tarsometatarsus of *L. titan* (GSI 3313) from Java. ei, eminentia intercotylaris; cl, cotyla lateralis; cm, cotyla medialis; fvd, foramina vascularia proximalia; fvp, foramina vascularia proximale; II, trochlea metatarsi II; III, trochlea metatarsi III; IV, trochlea metatarsi IV; se, sulcus extensorius. Image of *L. titan* courtesy of the Geological Museum Bandung.

Three tarsometatarsi (LB-Av-2476, -2479 and -2451) are the most complete elements known for *L. robustus* and represent at least two individuals. LB-Av-2476 (figure 12*a*) is a nearly complete right tarsometatarsus and is the largest of the three in all dimensions. Although it is broken in several places, its reconstructed length and all of its measurable dimensions extend well beyond that of extant *Leptoptilos* (table 9). The proximal end is damaged and distorted, but the eminentia intercotylaris is high, pointed, and mostly intact. The dorsal shaft surface is grooved by a distinct sulcus flexorius that extends to more than half its length. On the ventral surface, the proximal part of the hypotarsus is missing, but its vascular foramina lateral and medial are visible. The lateral foramen attains a more distal position than the medial one. The cristae plantares medialis and lateralis are distinct and extend toward the distal end. The shaft is squarish in cross-section with a thick cortical bone wall (up to approx. 3 mm). Distally, the three metatarsal trochlea are broken off and appear to have been flattened post-depositionally. The foramen vasculare distale is large, and the sulcus extending proximally from it on the dorsal surface measures approximately 13 mm. Trochlea metatarsi II and IV are equal in length, with trochlea metatarsi III extending further distally. Trochlea metatarsi III displays a distinct fovea, but the articular surface on trochlea metatarsi II and IV is smooth. The foramen vasculare distale is distinct and elongated.

LB-Av-2479 (figure 12*b*) is a complete left tarsometatarsus, with only the dorsal edge of the cotyla lateralis broken off. At 293 mm in length, it is shorter than LB-Av-2476 but similar to that of *L. dubius* and *L. crumenifer.* Its proximal end and shaft dimensions overlap with the largest specimens of *L. dubius*; however, the width and depth of its distal end are larger than in extant *Leptoptilos.* The bone surface appears fibrous with visible elongated pores, typical of juveniles. This bone surface structure, similar to ‘pattern C’ of Watanabe and Matsuoka [32], is visible along the whole shaft and extends to both the distal and proximal articular ends. At the proximal end in the fossa infracotylaris dorsalis and the surrounding surface, however, the bone surface structure is more open and loosely organized, and thin elongated ridges are visible with transverse struts, resembling ‘pattern B’ of Watanabe and Matsuoka [32]. There is no epiphyseal cartilage visible and the bone is fully ossified, although a suture is present proximally (figure 12*c*). This indicates that longitudinal growth of the tarsometatarsus had ceased but that circumferential growth was still ongoing. This combination of features is representative of fledglings, birds that have left the nest and are close to their adult size but are still in their first year. The eminentia intercotylaris is high and oriented laterally, with the top rounded and knob-like. In dorsal view, the cotyla medialis is narrower lateromedially than the cotyla lateralis. The fossa infracotylaris dorsalis is deep and contains two elongated foramina vascularia proximalia. The tuberositas m. tibialis cranialis is not developed yet as a distinct tuberosity, but the area distal from the foramina vascularia is rugose. The sulcus extensorius is distinct and continues long onto the shaft. On the caudal surface, the crista lateralis hypotarsi is slightly longer than the medial one (18.4 mm versus 14.1 mm). Two vascular foramina are visible on both sides of the cristae hypotarsi, but the lateral ones are located more distally than are the medial ones. The cristae plantares medialis and lateralis are less strongly developed than in the adult LB-Av-2476. Several superficial parallel rat gnawing marks are visible on the shaft. Distally, trochlea metatarsi III extends the furthest distally, and trochlea metatarsi II extends distally further than trochlea metatarsi IV. While the cranial surfaces of trochlea II and IV are relatively smooth, the trochlea metatarsi III has a distinct fovea and extends proximally until the base of the foramen vasculare distale. Caudally, all three trochleae metatarsorum display a distinct fovea. Specimen LB-Av-2451 consists of a shaft fragment only (figure 12*d*) that is similar in size to that of LB-Av-2479 but larger than in extant *Leptoptilos*. Its surface overall attains a generally smooth surface structure like that of LB-Av-2476, but it displays some patches of a fibrous texture. The dorsal surface of the shaft is distinctly grooved, similar to that of the other two specimens. The shaft flattens and splays toward the missing distal end. The shaft surface shows superficial rat gnawing marks.

#### 3.3.12. Phalanges

Four ungual phalanges (LB-Av-141, LB-Av-142, LB-Av-181 and LB-Av-185) and a right pedal phalanx 1 of digit II (LB-Av-164) are preserved. In all four ungual phalanges, the caput phalangis is only moderately curved. The tuberculum flexorium is set distally from the cotyla articularis and distinct sulci neurovasculares are visible on the medial and lateral sides of the corpus phalangis. The pedal phalanx is missing the medial facies of the distal half, but appears long and straight. Its estimated length (52 mm) overlaps with that of extant species (table 4 in [1]).

### 3.4. Taphonomy

Most *L. robustus* bones are incomplete and show minimal weathering, indicating that they spent only a short amount of time on the surface of the cave before they were buried. Some bones (e.g. right ulna LB-Av-3283) show considerably more weathering, such as thin longitudinal cracks and flaking of the bone surface that indicate longer exposure to the elements. This is further corroborated by several bones (e.g. LB-Av-2477) that show rodent gnawing marks (figure 8*c*). No Komodo dragon tooth marks or anthropogenic marks were observed on any of the stork bones.

### 3.5. Minimum number of *L. robustus* individuals represented at Liang Bua

**Figure 13.**
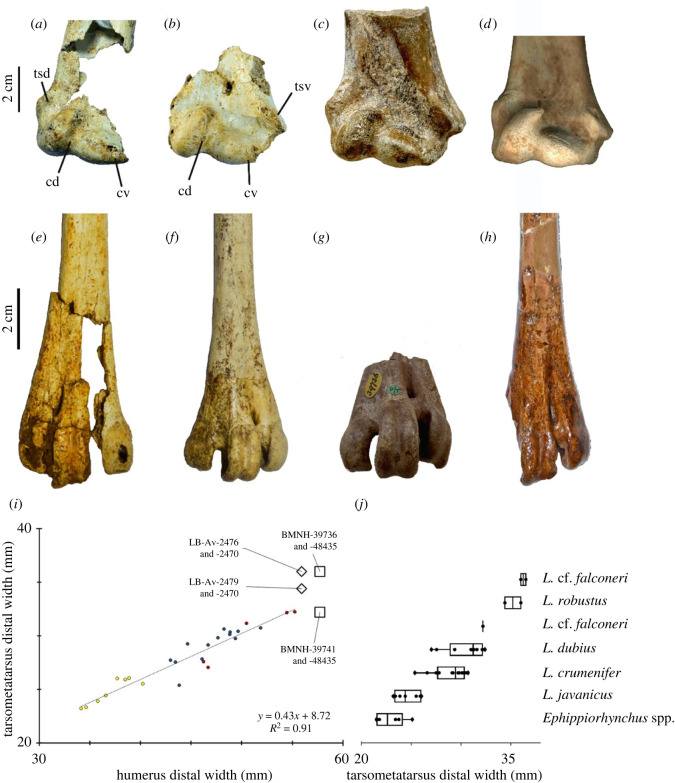
Humeri and tarsometatarsi of extinct species of *Leptoptilos.* (*a*), right humerus of *L. robustus* (LB-Av-179); (*b*), right distal humerus of *L. robustus* (LB-Av-2470); (*c*), mirror image of left distal humerus of *Leptoptilos falconeri* (NHMUK PV OR 48435) from the Siwalik Hills; (*d*), mirror image of the distal left humerus of *Leptoptilos lüi* (SAM 94. J. VIII-13.C-11) from Jinnishuan; (*e*), right tarsometatarsus of *L. robustus* (LB-Av-2476); (*f*), left tarsometatarsus of *L. robustus* (LB-Av-2451); (*g*), distal left tarsometatarsus of *L. falconeri* (NHMUK PV OR 39736) from the Siwalik Hills; (*h*), left tarsometatarsus of *L. titan* (GSI 3313) from Watoealang; (*i*), regression plot of humerus distal width against tarsometatarsus distal width for extant and extinct species of *Leptoptilos*; (*j*), box plot of tarsometatarsus distal width for extant and extinct species of *Leptoptilos* and *Ephippiorhynchus*. fvd, foramina vascularia distale; II, trochlea metatarsi II; III, trochlea metatarsi III; IV, trochlea metatarsi IV; se, sulcus extensorius. Images of *L. falconeri* courtesy of NHM, image of *L. lüi* courtesy of Z. Zhang, image of *L. titan* courtesy of the Geological Museum Bandung.

**Figure 14.**
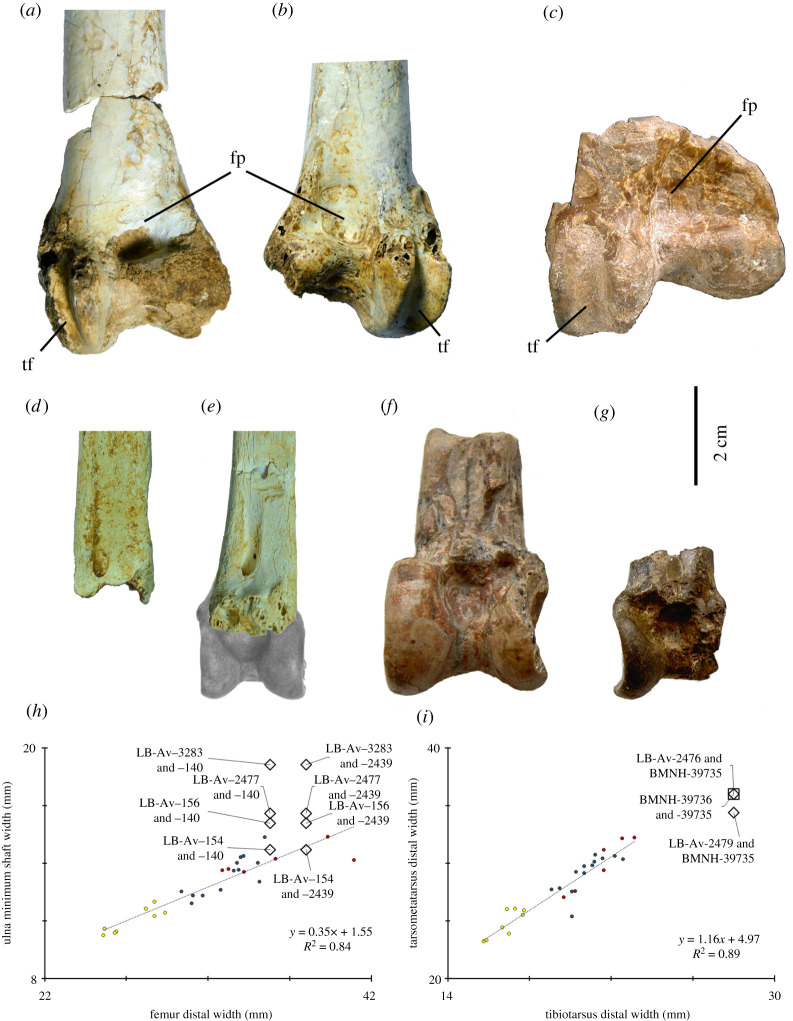
Femora and tibiotarsi of extinct species of *Leptoptilos.* (*a*), left femur of *L. robustus* (LB-Av-140); (*b*), right femur of *L. robustus* (LB-Av-2439); (*c*), distal left femur of *L. falconeri* (NHMUK PV OR 38737) from the Siwalik Hills; (*d*), distal left tibiotarsus of *L. robustus* (LB-Av-3360); (*e*), distal left tibiotarsus of *L. robustus* (LB-Av-155) with outline of estimated size of the distal end; (*f*), distal right tibiotarsus of *Leptoptilos falconeri* (NHMUK PV OR 39735) from the Siwalik Hills; (*g*), distal left tibiotarsus of *L. dubius/falconeri* (NHMUK PV OR 48444) from the Siwalik Hills; (*h*), regression plot of femur distal width versus ulnar minimum shaft width for extant and extinct species of *Leptoptilos*; (*i*), regression plot of tibiotarsus distal width against tarsometatarsus distal width for extant and extinct species of *Leptoptilos*. cd, condylaris dorsalis; cv, condylaris ventralis; fp, fossa poplitea; tf, trochlea fibularis; tsd, tuberculum supracondylare dorsale; tsv, tuberculum supracondylare ventral. Images of *L. falconeri* and *L. dubius/falconeri* courtesy of NHM.

**Figure 15.**
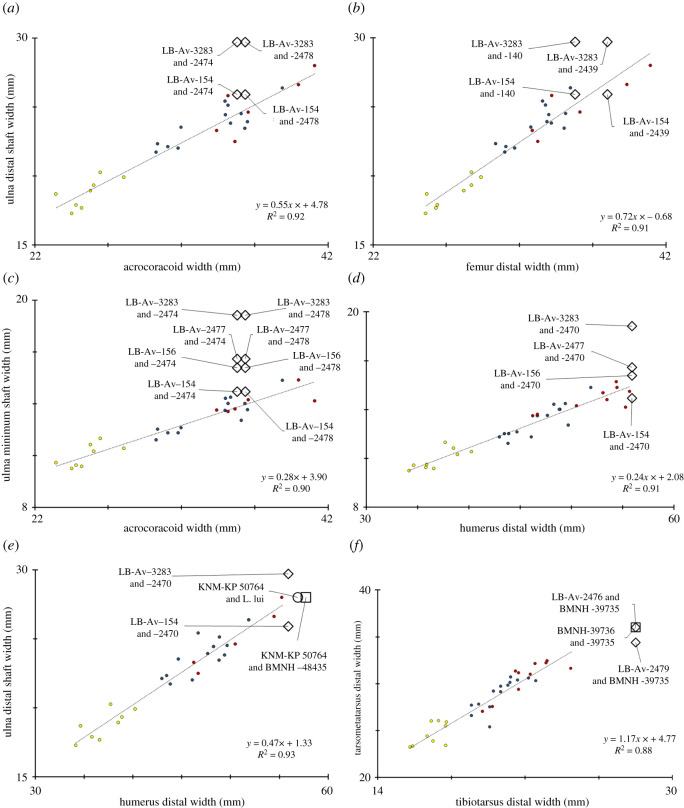
Bivariate comparisons of measurements from different elements in specimens of extant *Leptoptilos* species enable an assessment of whether different fossil elements represent specimens of similar size. (*a*), ulna distal shaft width against acrocoracoid width; (*b*), ulna distal shaft width against femur distal width; (*c*), ulna minimum shaft width against acrocoracoid width; (*d*), ulna minimum shaft width against humerus distal width; (*e*), ulna distal shaft width against humerus distal width; (*f*), tarsometatarsus distal width against tibiotarsus distal width.

Of the 43 *L. robustus* elements at Liang Bua, 38 are postcranial and of these, 18 have measurable dimensions that were quantitatively compared with extant and fossil *Leptoptilos*. Among the three extant species sample, bivariate comparisons of measurements from the same element (e.g. ulna proximal depth against ulna proximal width) and between elements (e.g. ulna minimum shaft depth against humerus distal width) show strong linear relationships (*R*^2^ values greater than 0.7) (e.g. figures 13–15). These relationships enable reasonable assessments of which *L. robustus* elements may belong to the same, or at least similarly sized, individuals. Two right coracoids (LB-Av-2474 and -2478), a left and right femur (LB-Av-140 and -2439), and a left ulna (LB-Av-154) are all proportionately smaller than expected compared with the other 13 measured elements (figures 14*h* and 15) and likely represent at least two small adult females, similar in size to *L. dubius* or larger *L. crumenifer* specimens. By contrast, a right tarsometatarsus (LB-Av-2476) is proportionately much larger than all the other elements and likely represents an adult male (figures 13*i*), which would have been considerably larger than any male *L. dubius*. Based on element duplication, the remaining 12 elements, all of which have comparably sized dimensions, likely represent at least two large female or perhaps small male individuals. Two elements—a left tibiotarsus (LB-Av-155) and a left tarsometatarsus (LB-Av-2479)—are not fully mature based on their bone surface textures [32,33]. However, the tibiotarsus and tarsometatarsus are typically among the last elements to mature skeletally [32] and this, in combination with the sizes of LB-Av-155 and -2479, means it is possible that both bones could be associated with one or both of these large female/small male individuals. Therefore, the total minimum number of *L. robustus* individuals represented at Liang Bua, based solely on the relative sizes of the 18 measurable specimens, is five: one large adult male, two large females or small males (one or both of which may not be fully mature), and two small adult females.

### 3.6. Comparisons with *Leptoptilos falconeri*

Fossil material attributed to *L. falconeri* [34] consists mostly of hindlimb elements with a few from the wing as well. A left distal humerus (NHMUK PV OR 48 435) from the Siwalik Hills in India was assigned to this species by Harrison [30] (using a previous registration number BMNH 48435). He measured 57.1 mm for its transverse width [30] whereas [35] previously reported 58.4 mm (2.3 inches). LB-Av-2470 preserves a transverse width of 50.9 mm but lacks approximately 5 mm due to damage and is thus similar in size (figures 6*d* and 13*i*). Harrison [30] suggested that a broad and deep groove between the attachment of the anterior articular ligament and internal (ventral) condyle as well as a large entepicondylar prominence and attachment of the anterior articular ligament were diagnostic characteristics of *L. falconeri*, and LB-Av-2470 also displays these features.

A distal right ulna (KNM-KP 50764) from the Pliocene of Kanapoi, Kenya was attributed to *L.* cf. *falconeri* by [3]. This specimen shows typical *Leptoptilos* morphology with a distinctive foramen between the condylus ventralis ulnae and the tuberculum carpale and is similar to the ulnae of *L. robustus.* Although there are no published measurements of KNM-KP 50764, an unpublished image (Field, personal communication, September 2021) yielded a distal width estimate of approximately 28 mm, which is within the size range of the two *L. robustus* ulnae (figure 8*f*). Two carpometacarpi, also attributed to *L.* cf. *falconeri*, are known from the Late Pliocene of Chad [1] and the Pliocene of Kenya [3], while another from the Early Pliocene of Ukraine [36] was assigned to cf. *L. falconeri* [1]. All of these share the foramen in the carpal trochlea, typical of all *Leptoptilos,* but the incomplete state of LB-Av-1 does not allow any meaningful comparisons. The paratype of *L. falconeri* is a distal left femur (NHMUK PV OR 39737) from the Siwalik hills. Although the specimen consists of only the articular end and sustains some damage, the preserved morphology is comparable to that of LB-Av-2439 and -140 although at 44.9 mm (1.77 inches) [35] its distal end is wider (figure 14*a–c*).

The holotype of *L. falconeri* is a distal end of a right tibiotarsus (NHMUK PV OR 39735) from the Siwalik hills [35]. In a revision of this taxon, Louchart *et al*. [1] attributed two other tibiotarsi to this species: KB3-97-161 from the Pliocene of Chad and OMO-122-76-367 from the Late Pliocene of Ethiopia. In addition, a tibiotarsus shaft (SAG-VP-1/19), a left distal tibiotarsus (URU-VP-1/28), and a right distal tibiotarsus (URU-VP-1/15), all from the Pliocene and Early Pleistocene of Ethiopia, were assigned to cf. *Leptoptilos falconeri* [1] while a left tibiotarsus (KNM-KP 56949) from the Pliocene at Kanapoi, Kenya was attributed to *Leptoptilos* cf. *falconeri* [3]. Unfortunately, LB-Av-155 and -3360 lack the distal articulation below the pons supratendineus, limiting comparisons with these other fossils. However, the minimum shaft widths and depths for the Liang Bua specimens are smaller than those of these others (figure 11*f*). LB-Av-3360 is slightly smaller than SAG-VP-1/19 and both Liang Bua specimens are perhaps comparable in size or slightly larger than NHMUK PV OR 48444, another specimen from the Siwaliks—originally described as *Cryptociconia indica* [30]—that Louchart *et al*. [1] attributed to *Leptoptilos dubius*/*falconeri* (table 8).

A distal end of a left tarsometatarsus from the Siwalik hills (NHMUK PV OR 39736) and an incomplete tarsometatarsus from Ethiopia (SAG-VP-1/19) are attributed to *L. falconeri* [1]. A left tarsometatarsus from Ukraine (IZAN 8024) that lacks its proximal end and a proximal fragment (KNM-KP 50800) from Kanapoi, Kenya [3] that includes articular surfaces are both considered *Leptoptilos* cf. *falconeri* [1]. Harrison [30] described that the trochlea metatarsi III extends more distally in *L. falconeri* than in extant species. However, as pointed out by Louchart *et al*. [1], this condition is also present in extant species. The minimum shaft widths of the Liang Bua tarsometatarsi (14.1, 12.4 and 12.1 mm) are similar to those reported for *L. falconeri* (13.2 mm) and *L.* cf. *falconeri* (13.2 mm) [1]. The first pedal phalanx is similar in morphology but slightly shorter than the *L. falconeri* pedal phalanx of digit II (F-516-23) from Omo Shungara, Ethiopia [1].

### 3.7. Comparison with *L. siwalicensis*

Harrison [30] re-examined the material referred to *L. falconeri* and attributed a left proximal tarsometatarsus (NHMUK PV OR 39741) and a right distal tibiotarsus (NHMUK PV OR 39734) to a new species *Leptoptilos siwalicensis* [30]. Harrison and Walker [37] also tentatively assigned a distal femur (NHMUK PV OR 11695) to this species. All three specimens were later referred to Leptoptilini gen. et sp. indet. by Louchart *et al*. [1]. Compared to the femora of *L. falconeri* and *L. robustus*, the distal femur NHMUK PV OR 11695 looks different: it is narrower, the crista tibiofibularis projects more distally and caudally than the trochanter fibularis, and the sulcus intercondylaris has a distinctly different shape. The lack of the distal articulation in the Liang Bua tibiotarsi hampers a direct comparison with the distal right tibiotarsus NHMUK PV OR 39734, but its shaft dimensions are slightly larger than those of the Liang Bua specimens (figures 11*f* and 14*i*). The proximal tarsometatarsus NHMUK PV OR 39741 appears broadly similar to that of LB-Av-2479, but its proximal width, the only measurement available [1], is below that of both LB-Av-2479 and LB-Av-2476.

### 3.8. Comparisons with *L. lüi*

A relatively complete cranium and a left distal humerus (SAM 94. J. VIII-13.C-11) as well as a proximal phalanx of the major digit of the wing (SAM 84.YJAT2-15) from the Middle Pleistocene of Jinniushan, China, are the holotype (cranium) and paratypes of *Leptoptilos lüi* [2]. Although the humerus was described as larger than that of *L. falconeri* (NHMUK PV OR 48435)—Zhang *et al*. [2] measured transverse widths of 56.9 and 52.1 mm, respectively—[35] reported a transverse width of 58.4 mm and Harrison [30] 57.1 mm for this same *L. falconeri* specimen. The estimated transverse width of LB-Av-2470 (approx. 56 mm) is thus in the size range of both *L. lüi* and *L. falconeri*, based on the measurements of [35] and Harrison [30]. Zhang *et al*. [2] also suggested that compared to *L. falconeri*, *L. lüi* has a narrower groove between the tuberculum supracondylare ventral and condylus ventralis as well, and the proximal tip of the condylus dorsalis points cranioventrally. In both Liang Bua humeri, the very proximal tip of the condylus dorsalis is damaged but the groove between the tuberculum supracondylare ventral and condylus ventralis appears wide like in *L. falconeri*.

### 3.9. Comparisons with *L. titan* and other stork material from Java

A large tarsometatarsus (GSI 3313) from Watualang on Java, Indonesia, is the holotype of *Leptoptilos titan* [4] (figure 12*f*). Although the age of Watualang is uncertain, it is generally considered part of the Ngandong faunal stage [38] and Late Pleistocene in age. An adult bone with a preserved length of 372 mm, it lacks the top of the eminentia intercotylaris; however, it is approximately 10 mm longer than the reconstructed length for LB-Av-2476. Comparison of *L. titan* with LB-Av-2476 is hampered by the significant damage to the latter's proximal end, but the shaft dimensions of *L. titan* are smaller than those of LB-Av-2476 and closer to those of the juvenile LB-Av-2479. Compared to LB-Av-2479 (figure 12*b*), the cotyla medialis of GSI 3313 is located more proximally than the cotyla lateralis, and in dorsal view, the cotyla medialis is rounded whereas in LB-Av-2479, it is elongated anterior-caudally. In addition, the cotyla lateralis protrudes more dorsally in *L. titan,* whereas in *L. robustus* the cotyla lateralis protrudes less, even though the exact degree of protrusion is difficult to establish due to the damage to the cotyla base. Distally, trochlea metatarsi III (albeit damaged at its distal end) projects beyond trochlea metatarsi II, a feature also observed in LB-Av-2479. The *L. titan* tarsometatarsus is longer and wider proximally compared to the immature LB-Av-2479 (which, although immature, shows a fusion of the epiphyses and is thus at its final length).

Three stork bones from the Middle Pleistocene Hauptknochenschicht (dated to between 0.54 ± 0.10 and 0.43 ± 0.05 Ma [39]), at Trinil, Java, were identified as *Leptoptilos* cf. *dubius* (a left ulna shaft, RGM.DUB.1491, and a left tibiotarsus shaft, RGM.DUB.1490) and *Ephippiorhynchus* cf. *asiaticus* (a proximal ulnar fragment, RGM.DUB.5913) [40]. Our sample of extant specimens shows that the dimensions of the proximal ulna fragment are on the lower end for *L. javanicus* and overlap with those of *Ephippiorhynchus.* Both the ulna and tibiotarsus shafts lack articular ends, but the overall morphology of these remains fits with that of Leptoptilini, including the Liang Bua specimens. The dimensions of the ulnar shaft RGM.DUB.1491 are just below that reported for the ulnae from Liang Bua and in the upper range of *L. dubius* and *L. crumenifer* (table 5). The tibiotarsus shaft RGM.DUB.1490 appears to belong to a juvenile, as elongated pores are visible on the bone surface. Its shaft dimensions are smaller than the Liang Bua tibiotarsi and overlap with those of *L. dubius* and *L. crumenifer* (table 8).

## 4. Discussion

Although *L. robustus* elements are extremely rare at Liang Bua, comprising less than 1% of the total faunal assemblage [18], together these remains represent one of the best samples in the world of an extinct giant marabou stork species. Stratigraphically concentrated in Unit 1B with two in Unit 3 (probably reworked from Unit 2 or possibly Unit 1B) (table 1), these giant stork elements represent at minimum one large adult male, two large females or small males (one or both of which may not be fully mature), and two small adult females. However, given that Unit 1B, which directly underlies a volcanic tephra (T1), spans approximately a 60 ka period (approx. 120–60 ka) [17], the actual number of storks represented is likely greater. These remains are associated with those of vultures (*Trigonoceps* sp.), dwarf proboscideans (*Stegodon florensis insularis*)*,* Komodo dragons and *Homo floresiensis* [14,18], all of which antedate the earliest evidence of modern humans (*Homo sapiens*) on Flores at approximately 46 ka [18]. Storks (and vultures) are not generally associated with caves, but these two large scavenging birds were likely dependent upon *Stegodon* as a source of carrion and attracted to carcasses in search of food [13,14]. With its shady overhang and recurring water pools (due to frequent flooding from the nearby river, the Wae Racang), Liang Bua likely was a comfortable shelter from the heat for local wildlife. Such a sheltered watering hole would have provided ample hunting and scavenging opportunities for Komodo dragons, *L. robustus, Trigonoceps* sp. and *H. floresiensis.* The fragmentary nature of the *L. robustus* assemblage suggests there may have been intense competition for *Stegodon* carcass access among these various taxa [19]. However, the *L. robustus* remains thus far do not show any signs of either Komodo dragon tooth marks or hominin butchery. Therefore, there is currently no evidence that *Homo floresiensis* played a role in the deaths of these individual storks or the extinction of this species.

The presence of two bones of osteologically immature birds, the LB-Av-2479 tarsometatarsus and the LB-Av-155 tibiotarsus, indicates that *L. robustus* was likely breeding in the area surrounding Liang Bua. These two specimens represent birds that had left the nest and were close to their adult sizes, but were still in their first year of life and likely sported immature plumage when they died. Breeding in storks is seasonal and based on breeding records for *L. javanicus*, the only extant species of *Leptoptilos* in Indonesia, limited to the dry season [41]. Normally about three eggs are laid and incubated by both sexes for about four to five weeks. The chicks are altricious and grow rapidly in the first few weeks. Fledging takes place after approximately 50 days in smaller stork species, but for larger species like the marabou stork, chicks fledge when they are around 90–110 days of age [42,43], which is an exceptionally long period [44] even though across Ciconiidae growth rate is negatively correlated with body mass [45]. At the time of fledging, chicks are comparable in size to, or even slightly heavier than, adult birds [32,43]. Given that *L. robustus* almost certainly had a larger average body mass than *L. crumenifer*, chicks of this extinct species likely fledged well after 110 days of age. Like most extant storks [46], *Leptoptilos* species are colonial breeders that build stick nests well off the ground in the tops of large trees. Large trees with broad canopies that are native to Flores include *Bischofia javanica* and *Terminalia* spp. (Y. Jehabut, personal communication, July 2021) and these would have been suitable for *L. robustus*. But was *L. robustus* able to fly?

When *L. robustus* was first described [8], it was suggested that its large size resulted from insular evolution (i.e. island gigantism) toward a more terrestrial lifestyle and a reduced flight ability because the LB-Av-154 ulna appeared smaller than expected given the dimensions of the LB-Av-155 tibiotarsus, which also appeared to have relatively thick cortical bone at its natural break along the distal half of the shaft. However, based on our analyses of the expanded hypodigm, which now includes more wing and hindlimb bones, as well as a pattern of relative bone sizes that is similar to that of extant *Leptoptilos* (figures 13–15), it is clear that LB-Av-154 and -155 represent individuals of different sizes. The LB-Av-3360 adult tibiotarsus also shows thinner cortical bone (2.5–2.8 mm) at its natural break that occurs slightly more distal along the shaft than in -155 (3.0–4.3 mm), which is also now recognized as juvenile. Cortical bone thickness may vary throughout the tibiotarsus shaft and/or the thicker bone wall in -155 might be due to its immature status [32]. Thus, whether tibiotarsus cortical thickness can be used as an indicator of locomotor behaviour in *Leptoptilos*, as suggested previously by Meijer and Awe Due [8], requires further study. The furcula, scapula, coracoid, humerus and ulna are all robust and well-developed and do not display any reduced skeletal proportions or osteological features commonly associated with flightlessness [47,48]. This suggests that *L. robustus* was capable of active flight, which has significant ecological implications.

**Figure 16.**
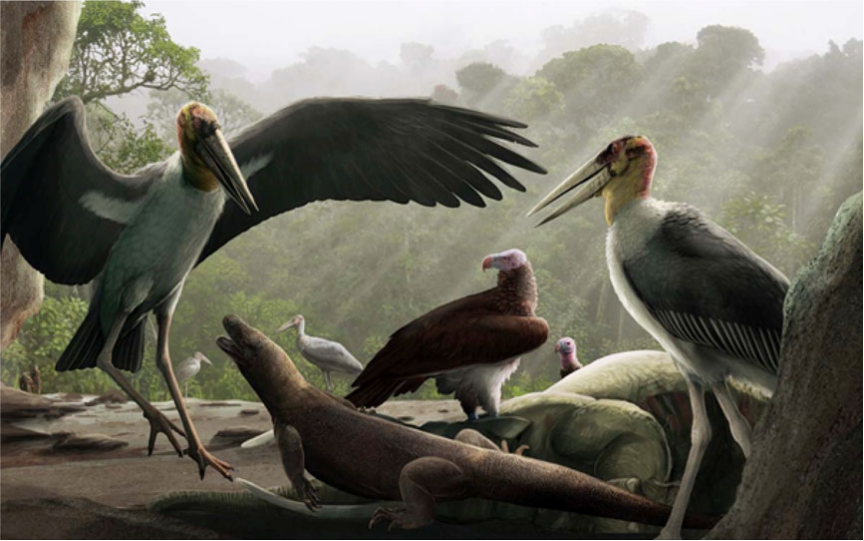
A possible scene at Liang Bua around 70 000 years ago. A giant marabou stork challenges a juvenile Komodo dragon for access to the carcass of a dwarf proboscidean while other giant marabou storks, vultures and hominins look on. Reconstruction by Gabriel Ugueto.

Large, broad-winged birds, such as raptors and storks, can and do use flapping (powered) flight for initial take-off and short distances. However, they generally avoid powered flight in favour of soaring-gliding flight. By exploiting rising air in the form of thermal currents (i.e. local columns of rising air generated by the sun) soaring-gliding flight is less energetically expensive [49]. Marabou storks rely heavily on thermal currents for flying and they can soar to great heights [46]. As the sun heated up open landscapes on Flores and thermals started to rise, *L. robustus* likely soared upwards with them. Flying at higher altitudes would have enabled these birds to sight prey from afar, see other conspecifics, like vultures, and/or Komodo dragons congregating in areas where food may have become temporarily available or abundant (figure 16). For stork colonies, food supplies close to their breeding sites often run out during the breeding season, at which times the birds will travel greater distances in search of food [46].

Soaring-gliding flight likely enabled *L. robustus* to reach Flores in the first place even though water is much less conducive to thermal formation than land. Soaring-gliding birds generally avoid long water crossings and instead tend to follow longer routes hugging coastlines rather than more direct over-water routes [49,50]. Large, open stretches of sea, such as the current Flores Sea north of Flores, were likely dispersal barriers for *L. robustus*; however, crossing the relatively narrow sea straits between the Lesser Sunda Islands, such as the approximately 19 km wide Sape Strait between Sumbawa and Flores, especially at times of low sea levels, would have been possible. At present, the Lesser Sunda Islands are part of a major migration pathway for many birds, including soaring-gliding raptors and wading birds. Known as the East Asian Continental Flyway, it stretches from the Lesser Sunda Islands to Southeast Asia to Eastern Siberia and includes several sea-crossings of 10–60 km [49]. Most large soaring-gliding birds cover these relatively narrow sea straits by either climbing to higher altitudes using thermals or by dynamic soaring where they make use of following winds [49]. Although extant *Leptoptilos* storks generally do not migrate, the Lesser Sunda Island chain was likely a main dispersal route into Wallacea for *L. robustus,* and probably also the vulture (*Trigonoceps* sp.) found at Liang Bua.

Given that *L. robustus* could almost certainly fly, its large size is therefore unlikely to have been the result of island gigantism, but rather a derived feature shared (i.e. a synapomorphy) with all species in the tribe Leptoptilini and the genus *Leptoptilos*, in particular [31]. For instance, *L. javanicus*, the smallest species within extant *Leptoptilos,* is large relative to most other birds, and the fossil record of Leptoptilini, which extends back into the Miocene [1], does not include any taxon smaller than this one. Indeed, osteologically the three extant *Leptoptilos* species differ from one another primarily in size (with considerable overlap between *L. crumenifer* and *L. dubius*) while limb bone proportions clearly distinguish *Ephippiorhynchus* from *Leptoptilos* [1]. The bones of *L. robustus* display an overall size range that overlaps but extends well beyond those of *L. crumenifer* and *L. dubius*. Morphologically, there is little doubt that *L. robustus* represents a large-bodied species of *Leptoptilos*. It has a furcula with a short, blunt, and mediolaterally broad projection of the extremitas sternalis, a foramen nervi supracoracoidei on the procoracoid (instead of an incisura nervi supracoracoidei as in all other storks), and a pneumatic foramen in the fovea carpalis caudalis of the carpometacarpus, all of which were retrieved as characteristic of a clade formed by *Leptoptilos* and the early Miocene species *Grallavis edwardsi* [31]. Moreover, the ventral portion of the coracoid's facies articularis ventralis clavicularis overhangs the ventral margin of the bone and the mandible appears dorsoventrally deep, both of which are features shared within *Leptoptilos.* However, *L. robustus* displays bone sizes and morphology that are broadly similar to *L. falconeri* remains from sites in Africa and Asia, and its overall size range is comparable to that implied by fossils attributed to *L. falconeri* and other similar specimens (e.g. *L*. cf. *falconeri* and cf. *L*. *falconeri*) as well as those of *L*. *lüi* (China) and *L*. *titan* (Java). These similarities raise important questions about the taxonomy and biological relationships of these geographically widely distributed, extinct giant marabou storks.

Given the apparent close affinities of *L. falconeri* and *L. robustus* in overall size and morphology, a hypothesis that *L. robustus* ancestry includes *L. falconeri* should be considered*.* In other words, dispersal of *L. falconeri* into Island Southeast Asia during the Pleistocene could explain the rise of local populations of giant marabou storks in this region: *L. robustus* on Flores as well as *L. titan* and *L*. cf. *dubius* on Java. A similar argument could be made with regard to *L. lüi*, which differs from *L. falconeri* in relatively minor morphological details (e.g. a narrower groove on the distal humerus [2]). Indeed, the best remaining distinguishing feature between the newly expanded sample of *L. robustus* and that of *L. falconeri* is that the latter may have had slightly smaller forelimbs compared with its hindlimbs [1] (e.g. compare figure 11 with figures 6 and 8). Thus, *L. robustus, L. titan, L*. cf. *dubius*, *L. lüi* and *L. falconeri* may represent either a single giant stork species or a group of very closely related species that stretched across Africa and Eurasia from the Pliocene until the Late Pleistocene. The close association of giant marabou storks with hominins, proboscideans and even vultures, at sites in Kenya [3], Chad and Ethiopia [1], northeastern China [2,51], Java [4], and Flores suggests that the dispersal of these birds into Island Southeast Asia probably happened as part of a larger scale faunal community dispersal tied to the expansion of drier, savannah-like ecosystems across the Sunda shelf during the Pleistocene [7,52,53]. Future findings of giant marabou stork and vulture remains in association with those of hominins and proboscideans from the region would confirm such a scenario, as would their absence from islands never colonized by proboscideans and/or hominins other than *Homo sapiens* (e.g. Timor) [54]. As the remains of *L. titan* and *L. robustus* appear to be the most recent representatives of these once plentiful giant marabou storks, Island Southeast Asia likely acted as a refugium for the last surviving members of these enigmatic birds.

## Data Availability

All data are included in the paper and the electronic supplementary material table [55].

## Appendix A. List of comparative specimens of *Leptoptilos* and *Ephippiorhynchus*

**Table:**

genus	species	institution	specimen number	sex
*Leptoptilos*	*crumenifer*	SMF	17772	F
*Leptoptilos*	*crumenifer*	SMF	1932	U
*Leptoptilos*	*crumenifer*	SMF	8249	F
*Leptoptilos*	*crumenifer*	SMF	2852	U
*Leptoptilos*	*crumenifer*	SMF	1933	U
*Leptoptilos*	*crumenifer*	BM	3094	U
*Leptoptilos*	*crumenifer*	NMNH	430819	F
*Leptoptilos*	*crumenifer*	NMNH	488128	M
*Leptoptilos*	*crumenifer*	NMNH	489396	M
*Leptoptilos*	*crumenifer*	NMNH	489395	F
*Leptoptilos*	*crumenifer*	NMNH	488129	F
*Leptoptilos*	*crumenifer*	NHM	S2014.65.1	M
*Leptoptilos*	*crumenifer*	NHM	1866.12.30.23	U
*Leptoptilos*	*crumenifer*	KUZM	228593	U
*Leptoptilos*	*javanicus*	NHM	S1952.3.131	U
*Leptoptilos*	*javanicus*	NHM	1850.8.15.62	U
*Leptoptilos*	*javanicus*	BM	3093	U
*Leptoptilos*	*javanicus*	NMNH	488758	U
*Leptoptilos*	*javanicus*	NMNH	223897	M
*Leptoptilos*	*javanicus*	NMNH	430764	U
*Leptoptilos*	*javanicus*	RBINS	1852B	U
*Leptoptilos*	*javanicus*	RBINS	12392	M
*Leptoptilos*	*javanicus*	KUZM	22.4.1889.2	U
*Leptoptilos*	*dubius*	NMNH	429220	F
*Leptoptilos*	*dubius*	NMNH	225988	F
*Leptoptilos*	*dubius*	NMNH	319788	M
*Leptoptilos*	*dubius*	RBINS	12394	U
*Leptoptilos*	*dubius*	RBINS	2101	U
*Leptoptilos*	*dubius*	RBINS	12395	M
*Leptoptilos*	*dubius*	RBINS	60379	F
*Leptoptilos*	*dubius*	KUZM	6.7.1896	U
*Leptoptilos*	*dubius*	KUZM	7.5.1938.1	U
*Leptoptilos*	*dubius*	KUZM	25.11.1915	U
*Leptoptilos*	*dubius*	KUZM	2.5.1899	U
*Ephippiorhynchus*	*senegalensis*	SMF	17732	U
*Ephippiorhynchus*	*asiaticus*	SMF	6338	U
*Ephippiorhynchus*	*asiaticus*	BM	3092	U
*Ephippiorhynchus*	*asiaticus*	NHM	1871.5.28.7	U
